# The soluble guanylate cyclase stimulator riociguat reduces fibrogenesis and portal pressure in cirrhotic rats

**DOI:** 10.1038/s41598-018-27656-y

**Published:** 2018-06-19

**Authors:** Philipp Schwabl, Ksenia Brusilovskaya, Paul Supper, David Bauer, Philipp Königshofer, Florian Riedl, Hubert Hayden, Claudia Daniela Fuchs, Judith Stift, Georg Oberhuber, Stefan Aschauer, Diana Bonderman, Thorsten Gnad, Alexander Pfeifer, Frank Erhard Uschner, Jonel Trebicka, Nataliya Rohr-Udilova, Bruno Karl Podesser, Markus Peck-Radosavljevic, Michael Trauner, Thomas Reiberger

**Affiliations:** 1 Division of Gastroenterology and Hepatology, Dept. of Internal Medicine III, Medical University of Vienna, Vienna, Austria; 2 Vienna Hepatic Hemodynamic Laboratory, Vienna, Austria; 3 Department of Pathology, Medical University of Vienna, Vienna, Austria; 4 Division of Cardiology, Dept. of Internal Medicine II, Medical University of Vienna, Vienna, Austria; 5 Institute of Pharmacology and Toxicology, University of Bonn, Bonn, Germany; 6 Department of Internal Medicine I, University of Bonn, Bonn, Germany; 7 Department of Gastroenterology, Odense Hospital, University of Southern Denmark, Odense, Denmark; 8 European Foundation of the Study of Chronic Liver Failure - EF CLIF, Barcelona, Spain; 9 Institute for Bioengineering of Catalonia, Barcelona, Spain; 10 Center for Biomedical Research, Medical University of Vienna, Vienna, Austria

## Abstract

In cirrhotic patients, portal hypertension (PHT) deteriorates survival, yet treatment options are limited. A major contributor to increased intrahepatic vasoconstriction in PHT is dysfunctional nitric-oxide signaling. Soluble guanylate cyclase (sGC) is the receptor of nitric-oxide and can be stimulated by riociguat. Riociguat is approved for pulmonary hypertension but has not been studied in liver cirrhosis. In this study we assessed the effects of riociguat on PHT and liver fibrosis in cholestatic (bile duct ligation, BDL) and toxic (carbon-tetrachloride, CCl4) rat models. In cirrhotic livers sGC expression was upregulated. In BDL rats, riociguat reduced liver fibrosis and decreased portal pressure without affecting systemic hemodynamics. In an early BDL disease stage, riociguat decreased bile duct proliferation, improved sinusoidal vascular dysfunction and inhibited angiogenesis. In advanced BDL riociguat exhibited anti-inflammatory effects. In CCl4 rats the beneficial effects of riociguat treatment were less pronounced and confined to an early disease stage. Similarly, in patients with cholestatic cirrhosis and PHT nitrates (that induce sGC activity) decreased portal pressure more effectively than in patients with non-cholestatic etiology. We also found an improvement of transaminases in patients with pulmonary hypertension receiving riociguat. Our findings support the clinical development of sGC stimulators in patients with cirrhotic PHT.

## Introduction

In liver cirrhosis intrahepatic vascular resistance is increased – causing portal hypertension (PHT)^1^. In turn, PHT may subsequently trigger development of hyperdynamic circulation^2^ and severe complications, such as variceal bleeding^3^ or ascites^4^. Non-selective betablockers (which reduce hepatic inflow)^5^ and nitrates (nowadays rarely used due to systemic side effects)^6^ are the only available medical treatments for PHT – but not all patients show a sufficient decrease of portal pressure^1^. Hence novel therapeutic targets^7^, such as the nuclear receptors PPARγ^8^ or FXR^9^, and the soluble guanylate cyclase (sGC) are currently explored.

Intrahepatic vascular resistance in cirrhosis is determined by both structural (i.e. fibrosis, vascular remodeling) and functional abnormalities (i.e. sinusoidal vasoconstriction, endothelial dysfunction)^10^. Endothelial dysfunction and sinusoidal vasoconstriction are driven by inflammation, oxidative stress and by an imbalance of vasodilators and vasoconstrictors. Nitric oxide (NO) represents the most important biogenic vasodilator, while in cirrhotic livers, both the production of and the response to NO are severely dysregulated^11^. The NO downstream signaling target sGC mediates vasodilation by catalyzing the reaction from GTP to cGMP^12^. The enzyme activity is predominantly regulated by a heme/Fe(II) group, which senses NO^12,13^. However, under conditions of oxidative stress, Fe(II) may be oxidized to Fe(III), thereby decreasing responsiveness to NO and thus deteriorating enzyme kinetics^14^. Pharmacologically, sGC activity can be increased using sGC stimulators such as riociguat (RIO)^15^. RIO targets sGC via an allosteric binding site and potentiates its sensitivity to low levels of bioavailable NO^16^. Direct modulation of sGC activity - downstream from NO - might be more beneficial than affecting NO production itself, since most detrimental effects of NO are cGMP-independent, while cytoprotective actions of NO are mediated via sGC^17^. Moreover, sGC stimulation by RIO may be more resistant against the negative cGMP-dependent protein kinase feedback loop^18^ or S-nitrosylation in stress conditions^19^, both limiting sGC activity.

In preclinical studies RIO has been shown to exert antihypertensive, antifibrotic and antiinflammatory effects, and to reduce vascular remodeling^20^. Recently, RIO has been approved for the treatment of pulmonary hypertension^21,22^. Two experimental studies investigated the effects of the sGC activator BAY 60–2770 in experimental cirrhosis: Knorr *et al*. demonstrated first, that BAY 60–2770 exhibits antifibrotic effects in rat models of CCl4-fibrosis and pig-serum induced liver injury^23^. Xie *et al*. confirmed these findings in a thioacetamide rat model and also observed an amelioration of sinusoidal architecture after BAY 60–2770 treatment^24^. Currently there are no data on the effects of sGC stimulation on PHT. However, prevention of cGMP degradation by phosphodiesterase-5-inhibitors (PDE5i), significantly reduced portal pressure (PP) in two clinical studies^25,26^. In line, PDE5i also reduced liver fibrosis, improved endothelial dysfunction and decreased PHT in cirrhotic rats^27,28^.

**Figure 1:**
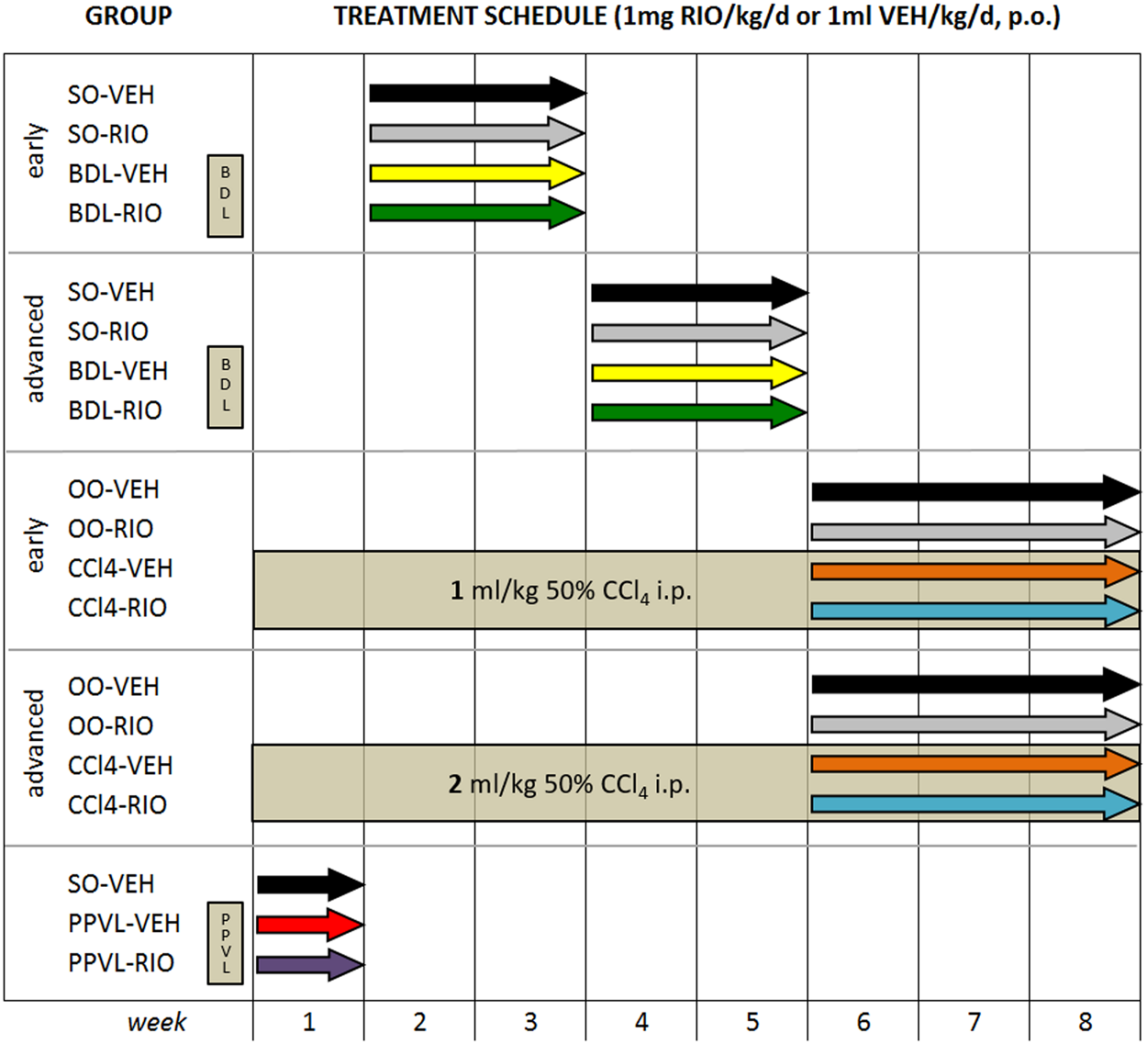
Study design and treatment groups. Riociguat or vehicle were gavaged once daily for 2–3 weeks to rats in early and advanced stages of cholestatic (BDL) or toxic (CCl4) cirrhosis, and to respective controls. Subsequently, portal and systemic hemodynamics were assessed and the degree of liver fibrosis quantified. The PPVL model was used to study hemodynamic effects in non-cirrhotic prehepatic portal hypertension.

Here, we investigate the effects of RIO on PHT and liver fibrosis in rats with early and advanced biliary (BDL) and hepatotoxic (CCl4) cirrhosis (Fig. 1). Furthermore, we aim to dissect the molecular mechanisms involved in RIO-induced modulation of sinusoidal vasotonus, angiogenesis, and inflammation.

## Results

### Riociguat ameliorates portal hypertension. The beneficial effects of sGC simulation are more pronounced in cholestatic cirrhosis than in toxic cirrhosis

All cirrhotic rats presented with significantly elevated PP compared to healthy controls and the degree of PHT increased with longer BDL duration (early BDL: 13.2 ± 2.5 mmHg, advanced BDL: 15.5 ± 1.6) and higher toxin exposure (early CCl4: 8.2 ± 0.9; advanced CCl4: 11.6 ± 2.5) (Table 1). In BDL rats, RIO significantly decreased PP in the early BDL (−24.1%; p = 0.048) as well as in the advanced BDL (−23.9%; p = 0.003) setting compared to vehicle (VEH) treated animals. Notably, RIO did not affect portosystemic shunting nor systemic hemodynamics. In the early toxic fibrosis model, RIO significantly decreased PP (−15.8%; p = 0.016), superior mesenteric artery blood flow (SMABF; −18.9%; p = 0.014) and tended to lower portosystemic shunting without deteriorating systemic hemodynamics. In contrast, in advanced CCl4 cirrhotic animals RIO did not exert beneficial effects on hepatic or systemic hemodynamics. Rats of the advanced CCl4 cirrhosis group presented an extensive disease with weight loss and death of five animals (n = 2 CCl4-VEH; n = 3 CCl4-RIO).

**Table 1:** Hemodynamics of early/advanced BDL and CCl4 rats.

		SO-VEH	SO-RIO	p BDL-VEH vs. SO-VEH	BDL-VEH	BDL-RIO	p BDL-VEH vs. BDL-RIO
BDL early	n	6	6		7	7	
	Weight (g)	374 ± 57	387 ± 27	0.733	366 ± 32	383 ± 35	0.311
	MAP (mmHg)	95 ± 20	109 ± 32	0.808	93 ± 10	104 ± 12	0.063
	Heart rate (bmp)	277 ± 25	304 ± 45	0.869	272 ± 60	321 ± 62	0.149
	SMABF (mL/min/100 g)	10.3 ± 4.0	10.2 ± 3.6	0.162	14.0 ± 4.1	9.8 ± 4.4	0.122
	Portal pressure (mmHg)	5.5 ± 1.1	6.4 ± 1.4	<0.001	13.3 ± 2.5	10.1 ± 2.4	0.048
	Shunting (%)	1.9 ± 1.8	2.1 ± 1.4	**0.092**	9.2 ± 8.4	9.5 ± 8.7	0.957
BDL advanced	n	5	5		8	8	
	Weight (g)	425 ± 29	433 ± 31	0.387	406 ± 39	409 ± 31	0.947
	MAP (mmHg)	111 ± 6	95 ± 16	0.164	99 ± 24	92 ± 24	0.351
	Heart rate (bmp)	363 ± 60	303 ± 61	0.241	319 ± 56	302 ± 46	0.584
	SMABF (mL/min/100 g)	9.3 ± 1.4	10.1 ± 2.1	0.005	14.3 ± 1.35	13.4 ± 2.4	0.498
	Portal pressure (mmHg)	6.6 ± 1.1	6.2 ± 1.5	<0.001	15.5 ± 1.6	11.8 ± 2.0	0.003
	Shunting (%)	1.39 ± 0.33	1.79 ± 1.30	**0.066**	19.0 ± 16.2	17.2 ± 15.1	0.680
		**OO-VEH**	**OO-RIO**	**p CCl4-VEH vs. OO-VEH**	**CCl4-VEH**	**CCl4-RIO**	**p CCl4-VEH vs. CCl4-RIO**
CCl4 early	n	7	6		7	6	
	Weight (g)	452 ± 30	468 ± 27	0.201	417 ± 43	412 ± 32	0.937
	MAP (mmHg)	96 ± 19	93 ± 12	0.356	84 ± 11	81 ± 10	0.626
	Heart rate (bmp)	307 ± 40	331 ± 29	0.017	264 ± 12	281 ± 34	0.212
	SMABF (mL/min/100 g)	8.3 ± 2.4	7.7 ± 1.3	<0.001	13.2 ± 1.5	10.7 ± 1.6	0.014
	Portal pressure (mmHg)	5.88 ± 0.89	5.50 ± 1.61	0.001	8.2 ± 0.9	6.9 ± 0.6	0.016
	Shunting (%)	1.15 ± 0.49	1.73 ± 1.50	0.001	25.2 ± 13.6	10.8 ± 6.77	**0.061**
CCl4 advanced	n	7	7		4	3	
	Weight (g)	449 ± 50	451 ± 38	**0.069**	364 ± 28	338 ± 31	0.089
	MAP (mmHg)	101 ± 26	113 ± 24	0.397	89 ± 22	112 ± 25	0.434
	Heart rate (bmp)	297 ± 47	323 ± 61	0.161	265 ± 99	305 ± 7	0.212
	SMABF (mL/min/100 g)	8.8 ± 1.4	9.7 ± 2.1	<0.001	19.3 ± 4.5	13.2 ± 3.0	0.204
	Portal pressure (mmHg)	5.1 ± 1.8	5.2 ± 0.8	<0.001	11.6 ± 2.5	11.2 ± 2.3	0.852
	Shunting (%)	2.1 ± 1.4	2.0 ± 1.9	<0.001	57.3 ± 24.4	39.5 ± 6.7	0.188

### Riociguat exerts antifibrotic activity in cholestatic and toxic models

**Figure 2:**
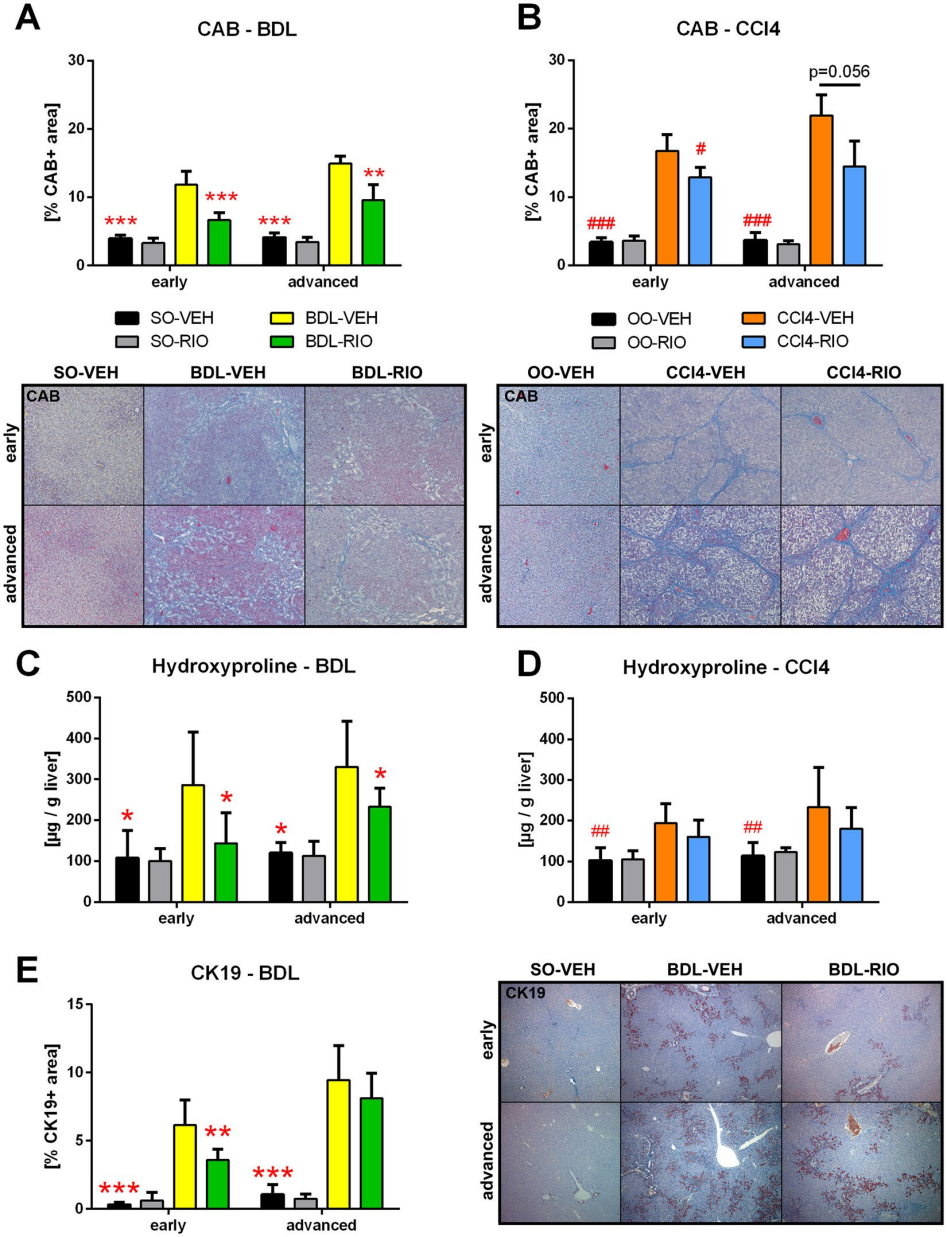
Riociguat exerts antifibrotic activity in cholestatic and toxic models. (**A**) Hepatic chromotrope-aniline-blue (CAB) stained area was quantified to assess fibrosis. In early and in advanced BDL rats, RIO significantly reduced CAB stained area. (**B**) In CCl4 cirrhosis RIO reduced CAB area only in early but not in advanced disease. (**C**) The liver fibrosis marker hydroxyproline content was measured photometrically and corrected to liver weight. RIO reduced hepatic hydroxyproline in both, early and advanced BDL rats. (**D**) No differences regarding hepatic hydroxyproline content were notable in early or advanced CCl4 animals receiving RIO. (**E**) Cytokeratin-19 (CK19) immunohistochemistry staining of liver slides were quantified to determine bile ducts. In early BDL-RIO rats less biliary proliferation was notable. Representative liver slides are shown in panel A, B and E. *p < 0.05, **p < 0.01, ***p < 0.001 vs. BDL-VEH, ^#^p < 0.05, ^##^p < 0.01, ^###^p < 0.001 vs. CCl4-VEH; two-sided unpaired t-test; n = 5–8 per group in panel A, C and D - according to Table 1; n = 3–7 per group in panel B and D - according to Table 1.

Both liver disease models presented with significantly increased hepatic fibrosis, as compared to healthy controls. A significant decrease in chromotrope-aniline-blue (CAB) stained area and hepatic hydroxyproline (HP) content was evident after RIO treatment in BDL rats with early (CAB: −44%; HP: −50%) and also advanced (CAB: −36%; HP: −29%) cholestatic cirrhosis (Fig. 2A–D). In the early cholestatic disease model, this effect was accompanied by less cytokeratin 19 (CK19) positive area in liver histology (−42%), indicating a reduction of ductular proliferation (Fig. 2E). In CCl4 rats, the antifibrotic effects were less pronounced. Only in the early CCl4 setting, a significant decrease of CAB stained area was detected.

### Riociguat favours intrahepatic vasodilation

**Figure 3:**
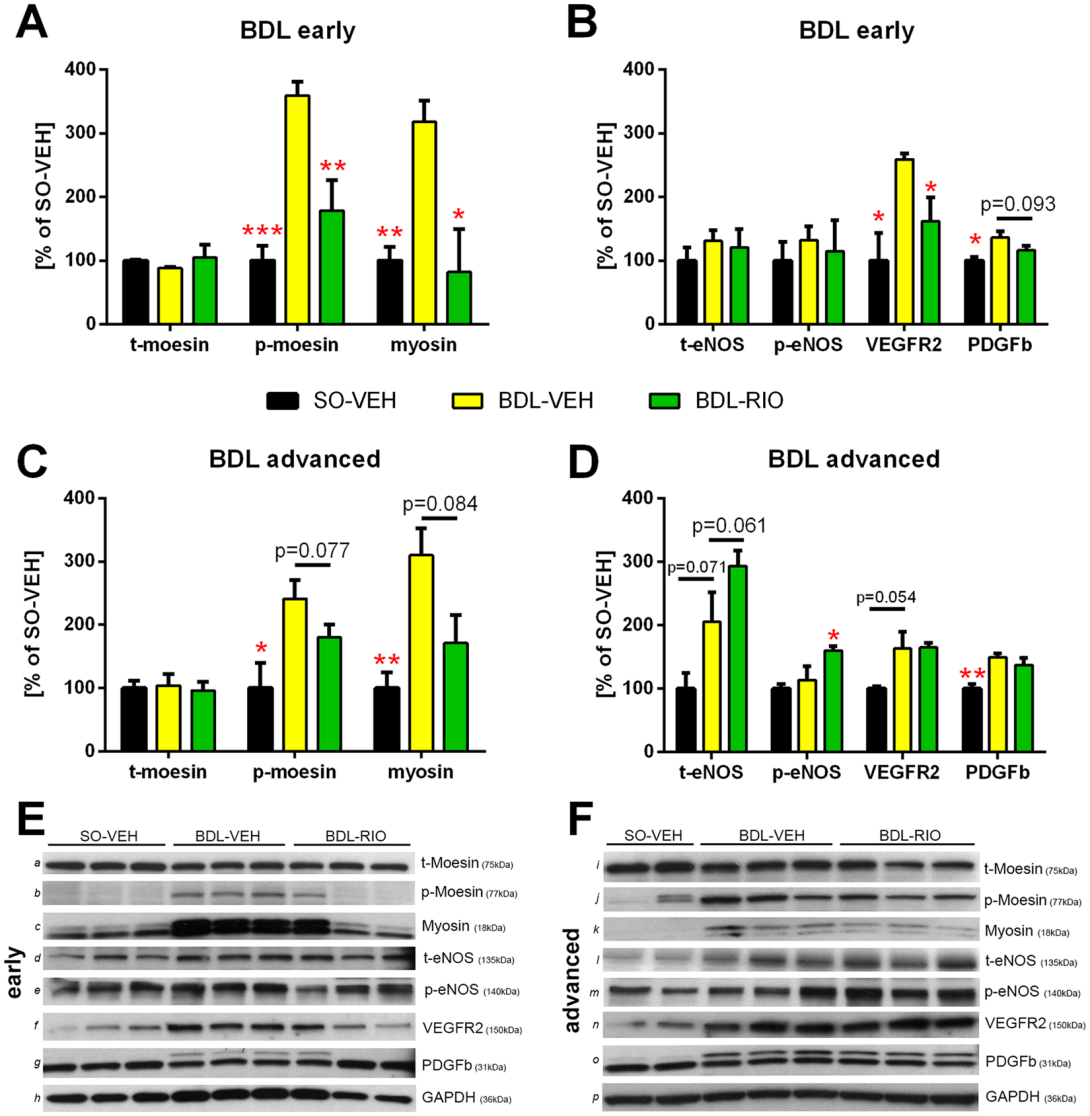
Riociguat improves intrahepatic vasodilation and vascular dysfunction. Western blots were performed to determine intrahepatic protein concentrations of markers of vascular contraction (total moesin [t-moesin], p-moesin, myosin), vascular dilation (t-eNOS, p-eNOS) and angiogenesis (VEGFR2, PDGFβ) in rats with (**A**,**B**) early and (**C**,**D**) advanced BDL cirrhosis. Values were normalized to expression of glyceraldehyde 3-phosphate dehydrogenase (GAPDH) as housekeeping protein. (**A**) BDL significantly increased hepatic moesin phosphorylation and myosin content in rats with early BLD cirrhosis, which was counter-regulated by RIO treatment. (**B**) VEGFR2 and PDGFβ expression increased significantly in early BDL rats, whereas after RIO treatment expression of both remained low. (**C**) RIO tended to normalize moesin phosphorylation and myosin content in rats with advanced BDL. (**D**) While eNOS protein content and phosphorylation were increased by RIO therapy, VEGFR2 and PDGFβ expression remained unchanged in advanced BDL-RIO animals. (**E**,**F**) Representative Western blots of early [*a-h*] and advanced [*i-p*] BDL animals. Full-length blots are presented in Supplementary Figure S5. *p < 0.05, **p < 0.01, ***p < 0.001 vs. BDL-VEH; two-sided unpaired t-test; n = 3 per group in panels A, C, E n = 2-3 per group in panels B, D, F.

In early cholestatic (BDL) cirrhosis, western blot analysis of vasoactive proteins expression revealed a strong reduction of moesin phosphorylation (p-moesin) and myosin light chain production upon treatment with RIO (Fig. 3A). This was accompanied by a decrease of intrahepatic vascular endothelial growth factor receptor 2 (VEGFR2) and platelet derived growth factor beta (PDGFβ) expression, while levels of endothelial nitric oxide synthase (eNOS) did not change (Fig. 3B). In BDL-RIO rats with advanced cirrhosis, there was still a trend towards less moesin phosphorylation and myosin expression notable (Fig. 3C). However the increased expression of VEGFR2 and PDGFβ after bile duct ligation remained unchanged upon RIO treatment. In contrast, in advanced BDL rats RIO caused an increase in total (t-eNOS) and phosphorylated eNOS (p-eNOS) (Fig. 3D). Yet, intrahepatic NO_x_ levels were not significantly affected by RIO - neither in early nor in advanced stage of cholestatic/BDL cirrhosis (Supplementary Fig. S1).

Notably, also in rats with early CCl4 cirrhosis RIO significantly decreased hepatic moesin phosphorylation (p-moesin) and myosin light chain production. Yet in this group RIO had no significant effect on eNOS, VEGFR2 or PDGFβ expression (Supplementary Fig. S2A,B).

### Riociguat reduces hepatic inflammation

**Figure 4:**
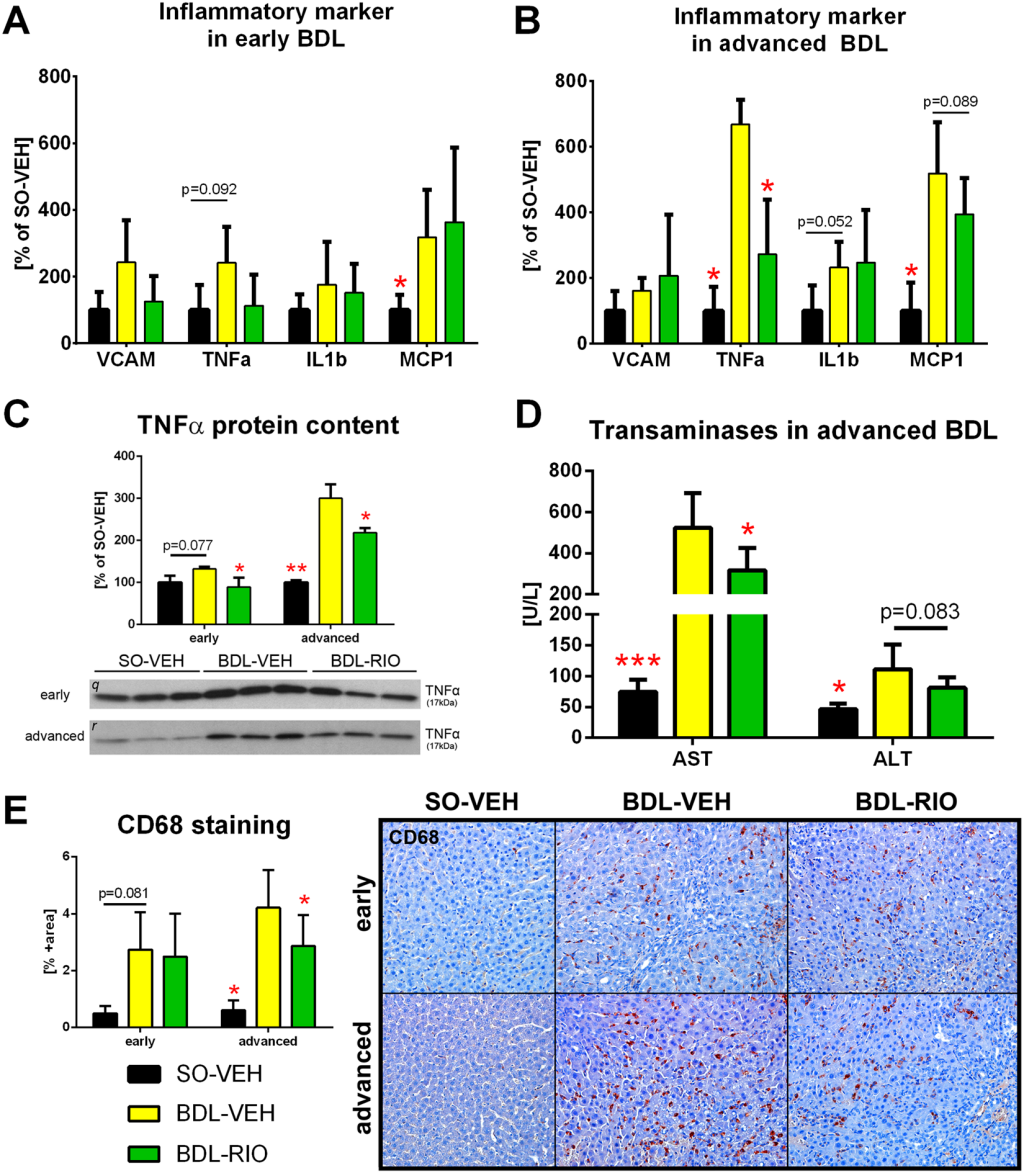
Riociguat reduces hepatic inflammation. RT-PCR was performed in liver tissue to screen for expression changes of inflammatory marker. (**A**) In the early BDL model expression differences were mostly non-significant, when compared to SO-VEH. Yet, in RIO treated animals mean VCAM and TNFα were decreased by 48.5% and 46.7%, respectively. (**B**) In advanced BDL animals, RIO significantly decreased TNFα and tended to reduce MCP1 expression. (**C**) Western Blotting was used to measure hepatic TNFα protein content, which significantly decreased in BDL rats receiving RIO. (**D**) Transaminases AST and ALT were measured in serum samples. RIO caused a significant decrease of AST and a trend towards lower ALT levels in rats with advanced BDL. (**E**) Hepatic immunohistochemistry stainings of CD68 positive cells were quantified to assess macrophage infiltration. In advanced BDL rats CD68 positive cell content was significantly decreased after RIO treatment. Full-length blots of the cropped lines [*q, r*] are presented in Supplementary Figure S5. *p < 0.05, **p < 0.01, ***p < 0.001 vs. BDL-VEH; two-sided unpaired t-test; n = 5–8 per group in panel A, B, D and E - according to Table 1; n = 3 per group in panel C.

BDL causes hepatic inflammation and induced expression of the proinflammatory cytokines vascular cell adhesion protein 1 (VCAM), tumor necrosis factor alpha (TNFα), interleukin 1 beta (IL1β) and monocyte chemoattractant protein 1 (MCP1). In the early BDL model the expression differences of these biomarkers were mostly non-significant, when compared to SO-VEH. Thus also the impact of RIO treatment attained no statistical significance, even though mean VCAM and TNFα expression were decreased by 48.5% and 46.7%, respectively (Fig. 4A).

However, in advanced cholestasis, RIO treatment resulted in a significant decrease of TNFα mRNA and tended to reduce MCP1 expression, while VCAM and IL1β remained unchanged (Fig. 4B). To confirm this signal, hepatic TNFα protein content was measured and indeed RIO normalized the upregulation of hepatic TNFα in BDL rats (Fig. 4C). Ultimately, BDL-RIO rats also showed reduced serum levels of aspartate transaminase (AST; −39%) and alanine transaminase (ALT; −27%) as compared to BDL-VEH animals (Fig. 4D). To assess the impact on hepatic macrophage infiltration, cluster of differentiation 68 (CD68+) was stained in liver slides. In line with the previous observations, RIO treatment significantly decreased CD68+ area in rats with advanced cholestasis (Fig. 4E).

### Riociguat inhibits the fibrogenic phenotype of hepatic stellate cells *in vivo* and *in vitro*

**Figure 5:**
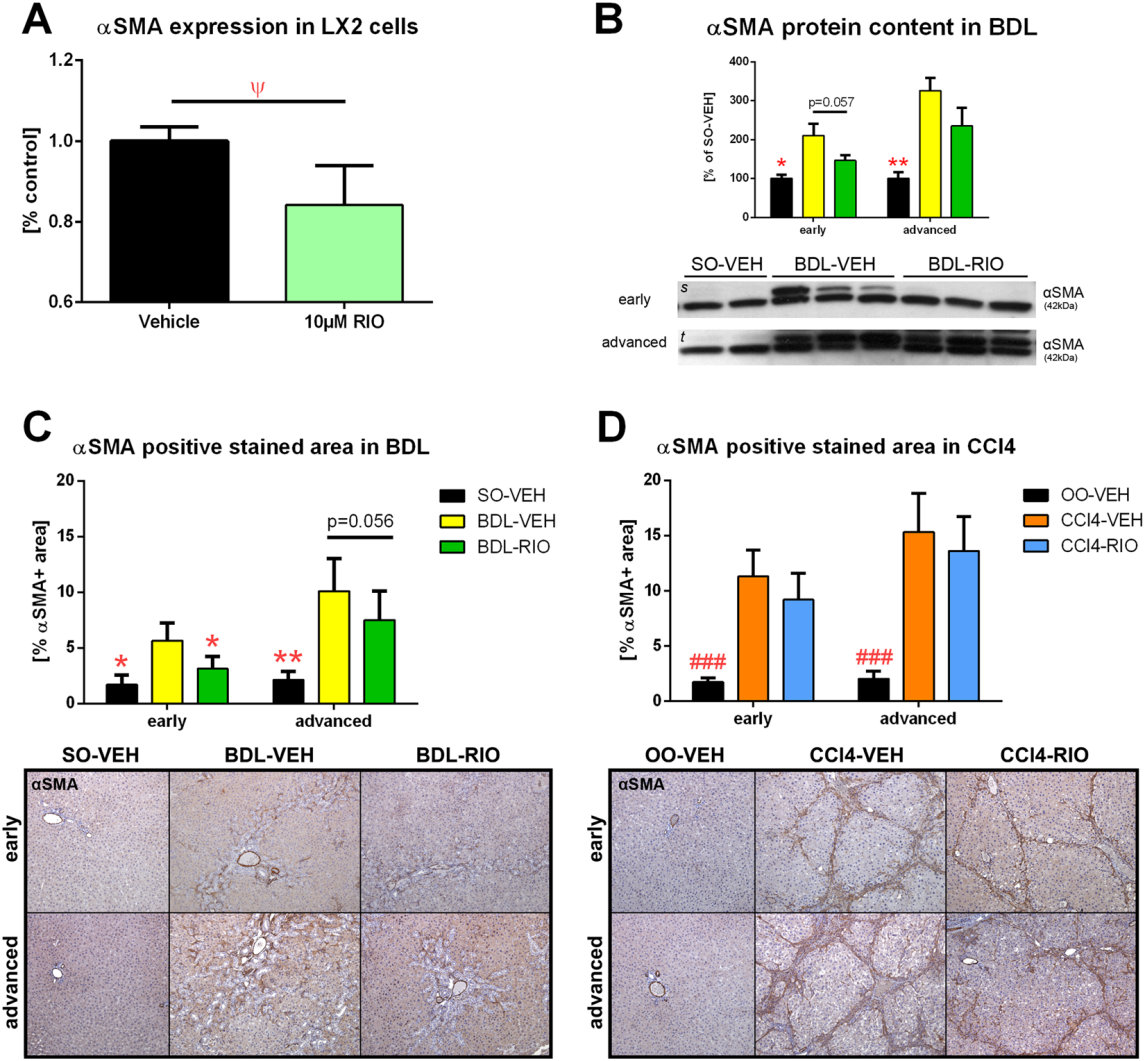
Hepatic stellate cell activation *in vitro* and *in vivo*. (**A**) Cultured LX-2 HSCs were treated for 48 h with 10 µM RIO or vehicle, followed by RT-PCR of αSMA mRNA expression. RIO caused a mild but significant decrease in αSMA expression compared to controls. (**B**) Hepatic αSMA protein content was measured by western blotting. It tended to be lower in early BDL rats treated with RIO, while no significant changes were seen in rats with advanced BDL. (**C**) The αSMA positive stained area of liver slides from BDL rats was lower in the RIO group, attaining statistical significance in the early setting and also showing a decreasing trend in the advanced disease model. (**D**) Yet, hepatic αSMA stainings from CCl4 cirrhotic rats showed no significant changes after RIO treatment. Full-length blots of the cropped lines [*s, t*] are presented in Supplementary Figure S5. ^Ψ^p < 0.05 10 µM RIO vs. vehicle control; *p < 0.05, **p < 0.01, ***p < 0.001 vs. BDL-VEH, ^#^p < 0.05, ^##^p < 0.01, ^###^p < 0.001 vs. CCl4-VEH; two-sided unpaired t-test; n = 6 per group in panel A; n = 2-3 per group in panel B; n = 5–8 per group in panel C - according to Table 1; n = 3–7 per group in panel D - according to Table 1.

To further investigate the molecular effects of RIO, LX-2 HSCs were treated with RIO, where a significant decrease of alpha smooth muscle actin (αSMA) gene expression was observed (Fig. 5A). Since αSMA is mainly expressed in HSCs, hepatic αSMA content was quantified by Western Blot and αSMA-positive area was quantified *in vivo* by histological analysis. In BDL rats with early cirrhosis, RIO treatment decreased intrahepatic αSMA protein expression and αSMA positive stained area in liver slides (5.66 ± 2.43 vs. 3.12 ± 1.92%; p = 0.013) (Fig. 5B,C). We also observed a non-significant decrease in hepatic αSMA protein content and a trend towards lower αSMA expression in liver histology of BDL-RIO rats with advanced cirrhosis (Fig. 5B,C). Yet, in CCl4 rats only minor changes of hepatic αSMA were detected, as quantified in liver histology (Fig. 5D) and by protein content (Supplementary Fig. S2C,D).

### sGC is expressed in the liver and upregulated in BDL rats

**Figure 6:**
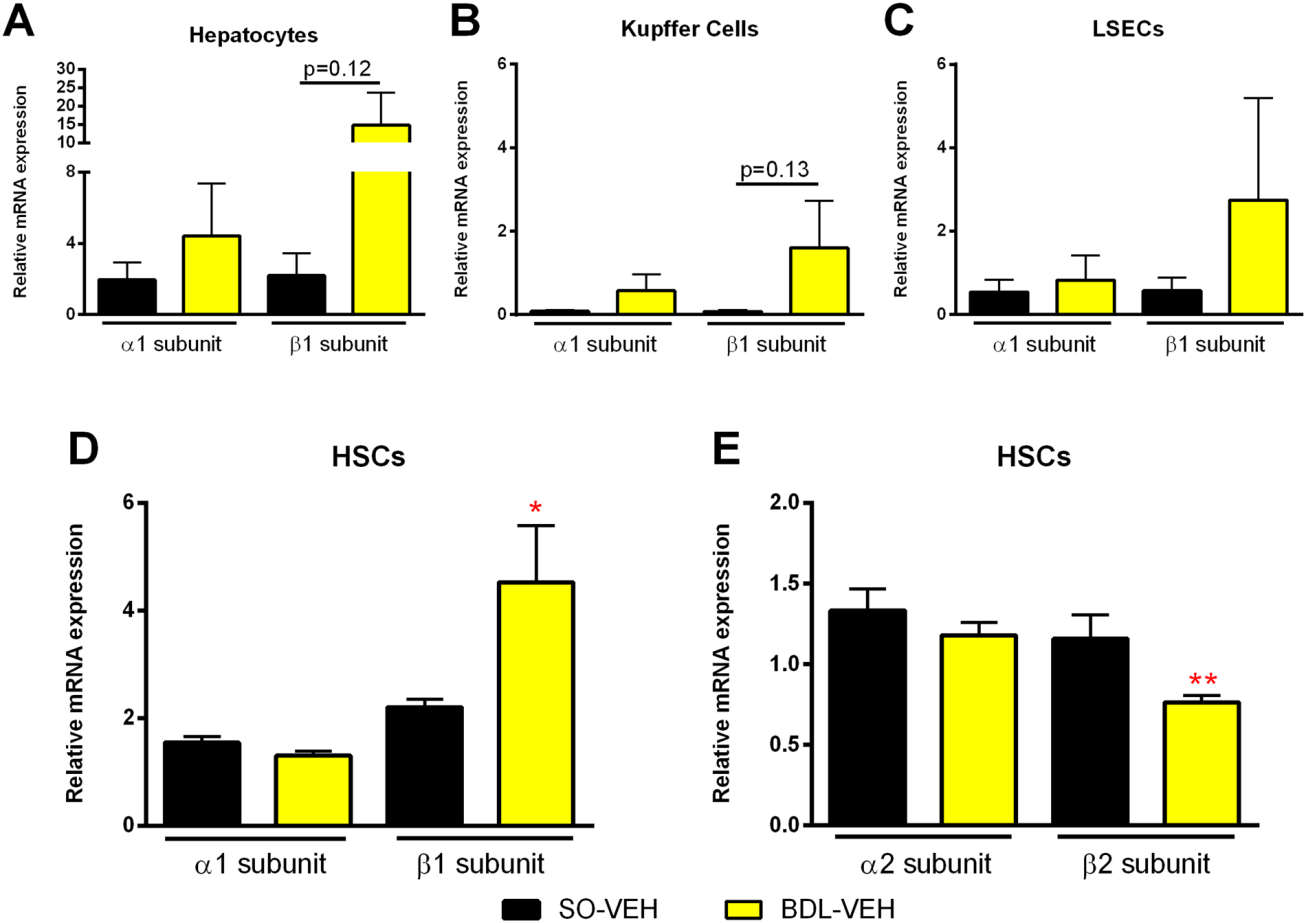
Hepatic sGC expression is upregulated in BDL rats. Subsets of liver cells were extracted from healthy and BDL rats. sGC subunit expression was analysed using RT-PCR. (**A**,**B**) In hepatocytes and Kupffer cells BDL animals presented a trend towards increased β1 subunit expression. (**C**) No significant changes were notable in LSECs. (**D**,**E**) Hepatic stellate cells presented a significant increase of sGCβ1 expression, paired with a reduction of the less active sGCβ2. *p < 0.05, **p < 0.01, ***p < 0.001 vs. BDL-VEH; two-sided unpaired t-test; n = 5–6 in all panels.

In healthy rat livers, we detected expression of sGC subunits α1 and β1 mostly in hepatocytes and hepatic stellate cells (HSC), but also in liver sinusoidal endothelial cells (LSEC) and to a very low extent in Kupffer cells (Fig. 6A–D). In BDL cirrhotic animals, we measured a significant upregulation of the sGC β1 subunit in HSCs (Fig. 6D) and additionally a trend towards increased sGC expression in other liver cell subsets. In contrast, α1 expression remained unchanged. We thus further investigated the impact of BDL on expression of the less common α2 and β2 subunits in HSCs. Here we noted a significant downregulation of the β2 subunit, while again expression of the α2 subunit was unaffected by cholestasis (Fig. 6E).

### Riociguat decreases portal pressure in prehepatic portal hypertension, but at the cost of increased portosystemic shunting

In a non-cirrhotic portal hypertensive partial portal vein ligation (PPVL) model, RIO also significantly decreased PP (12.7 ± 1.6 vs. 10.7 ± 0.9 mmHg; p = 0.025) (Table 2). However, this effect was accompanied by a significant decrease in mean arterial pressure (−16.7%; p = 0.045) and an increase in portosystemic shunting (+68.5%; p = 0.007) (Table 2).

**Table 2:** Hemodynamics of PPVL rats.

	SO-VEH	p SO-VEH vs. PPVL-VEH	PPVL-VEH	PPVL-RIO	p PPVL-VEH vs. PPVL-RIO
n	5		7	7	
Weight (g)	351 ± 13	0.305	338 ± 20	335 ± 14	0.714
MAP (mmHg)	92 ± 8.1	0.634	87 ± 6.3	75 ± 9.5	0.045
Heart rate (bpm)	353 ± 23	0.128	327 ± 26	314 ± 37	0.734
SMABF (mL/min/100 g)	4.1 ± 1.8	<0.001	11.7 ± 2.2	14.0 ± 1.6	0.446
Portal pressure (mmHg)	6.5 ± 1.0	0.005	12.7 ± 1.6	10.7 ± 0.9	0.025
Shunting (%)	8.5 ± 3.9	0.011	45.9 ± 20.1	77.5 ± 13.7	0.007

### Riociguat reduces serum levels of transaminases and alkaline phosphatase in non-cirrhotic patients

Since RIO is available for treatment of pulmonary hypertension, we studied the individual time course of transaminase levels in 27 non-cirrhotic patients with pulmonary hypertension and associated heart failure with preserved ejection fraction (PH-HFpEF) before, at baseline, during and after treatment with RIO (Supplementary Fig. S3A, Supplementary Table S1). Notably, during RIO treatment a significant decrease of AST (−15%), ALT (−10%) and alkaline phosphatase (AP; −7%) was observed, while levels of gamma-glutamyl transferase (GGT) remained unaffected (Supplementary Fig. S3B–E). Of note, in the time period prior to treatment initiation we did not observe any significant changes, and in a subgroup of patients who discontinued RIO (n = 13) the respective values returned back to baseline. We further studied the time course of transaminases, AP and GGT in age-matched, non-cirrhotic PH-HFpEF patients receiving standard medical treatment (n = 34). This control group had similar baseline parameters (Supplementary Table S1) and presented no significant changes of AST, ALT, AP or GGT after treatment initiation (Supplementary Fig. S3F–I).

### Cirrhotic patients with cholestatic liver disease show more pronounced decreases in portal pressure in response to NO donors than patients with non-cholestatic etiologies

In the animal studies, BDL rats consistently presented superior results with RIO as compared to CCl4 animals. To further investigate a potential etiology-dependent impact of the NO pathway on PHT, we retrospectively reviewed hemodynamic response rates of 56 cirrhotic patients undergoing repetitive hepatic venous pressure gradient (HVPG) measurements prior and under NO-donor (nitrate) therapy. The patients were subdivided in cholestatic or non-cholestatic etiologies of liver disease (Supplementary Fig. S4A). The baseline characteristics between these two groups were similar, except for sex and bilirubin content (Supplementary Table S2). Indeed, patients with cholestatic cirrhosis (n = 7) showed a significantly higher rate of HVPG response to nitrates (86% vs 43%; p = 0.034) compared to patients with alcoholic liver disease or viral hepatitis (n = 49). This was also underlined by a trend towards a greater mean HVPG decrease in cholestatic liver disease (−22.1% vs. −9.9% in non-cholestatic disease; p = 0.092) (Supplementary Fig. S4B–E).

## Discussion

Impairment of the NO/sGC/cGMP pathway represents a major determinant of the increased intrahepatic vascular resistance in patients with cirrhosis, and thus is a promising target for the treatment of portal hypertension^11^. Here, we show that direct sGC stimulation by RIO does not only decrease portal pressure, but also reduces hepatic inflammation and liver fibrosis. The beneficial effects of RIO were most pronounced in rats with cholestatic (BDL) cirrhosis and in early toxic (CCl4) cirrhosis.

Most importantly, we observed clinically relevant decreases of portal pressure in early CCl4 cirrhosis (−16%), in early cholestatic (−24%) and advanced cholestatic (−24%) cirrhosis without significant effects on mean arterial pressure. Moreover, in cirrhotic animals RIO did not affect splanchnic blood flow or portosystemic shunting, suggesting that the vasodilatory effects of sGC stimulation seem to prevail in the damaged hepatic sinusoids. Of note, previous studies using NO donors^29,30^ or PDE5i^28,31^ (which act up- and downstream of sGC) have led to conflicting results regarding amelioration of PHT. Lack of intrahepatic specificity and less antifibrotic activity might be a reasons for these inconclusive observations. While NO donors are indeed vasodilatory drugs, NO itself also leads to detrimental side-effects via other pathways, thus promoting inflammation, HSC apoptosis or even liver fibrosis^32,33^. Furthermore, in cirrhotic HSCs, NO donors failed to increase cGMP production and thus vasodilation^34^, probably because long-term NO exposure decreases sGC mRNA stability^35^. PDE5i are also potent vasodilators, yet the hepatic PDE5 expression is weak as compared to other organs^36,37^.

In contrast to NO the sGC pathway focuses primarily on vasodilation, and in contrast to PDE5, the sGC enzyme is highly expressed in the liver, especially in HSCs and portal venules^38^. This is supported by our novel finding, that after BDL hepatic sGC expression is further upregulated, particularly in HSCs, hepatocytes and Kupffer cells. Of note, we observed in HSCs of cholestatic BDL animals not only an increased expression of the more active β1 subunit, but also downregulation of the futile β2 subunit. This expression shift particularly supports production of the two most active sGC isoforms α2β1 and α1β1^39^. The observed intrahepatic sGC expression changes might occur due to a lack of NO^40^ and deteriorated oxygen metabolism^41^. Additionally this might explain, why sGC stimulation exerts its vasodilatory effects mainly in the cirrhotic intrahepatic microcirculation.

In the non-cirrhotic PPVL model RIO also decreased portal pressure, yet this occurred through other mechanisms, since in these animals the liver architecture is not altered. In PPVL rats we observed a concomitant reduction in systemic arterial pressure and an increase in portosystemic shunting. This suggests that in absence of cirrhosis, vasodilatory effects are more apparent on the systemic, splanchnic and collateral vasculature. In addition, the high degree of portosystemic shunting (typical for the PPVL model) decreases the hepatic first pass effect and leads to increased systemic exposure of RIO. Furthermore, due to the fixed prehepatic surgical portal stenosis, intrahepatic vasodilation is ineffective to decompress the portal system. Moreover, in PPVL animals sGC stimulation might have a higher impact of on the systemic circulation, because arterial cGMP levels remain normal (and thus are more susceptible to sGC stimulation), whereas in cirrhosis arterial cGMP levels are already upregulated^42^. Ultimately the RIO-treatment induced increase in shunting and reduction of mean arterial pressure in PPVL rats are hemodynamic safety signals, which may limit its use in non-cirrhotic portal hypertension.

The key factor contributing to cirrhotic PHT is increased intrahepatic vascular resistance, which is caused by both matrix deposition (fibrosis) and sinusoidal vasoconstriction^10^. Notably, we could demonstrate that RIO exerts beneficial effects not only on the sinusoidal vascular tone but also on liver fibrosis. In line with previous reports^23,24^, we observed a significant reduction of liver fibrotic CAB stained area, decreased hydroxyproline content and less αSMA-positive myofibroblasts in cirrhotic rats treated with RIO. Additionally, we confirmed that RIO also reduces hepatic stellate cell derived αSMA expression^24^. In order to decipher the anti-fibrotic potential of RIO, we used two rat models of toxic and cholestatic cirrhosis, and moreover studied them at two disease stages, since antifibrotic effects are more difficult to achieve in advanced cirrhosis. While in the toxic CCl4 model benefits of RIO treatment were limited to early cirrhosis, in BDL rats we observed significant improvements of PHT and liver fibrosis also in the advanced disease model. The particular effects of RIO in the cholestatic BDL model were – at least partly – mediated through a reduction of bile duct proliferation as shown by a decreased CK19 expression. The underlying cellular interplay and effects on cytokine expression have been comprehensively described by Xie *et. al*.^24^, who showed that sGC activation normalizes the LSEC phenotype and thereby promotes HSC quiescence. Thus, sGC activation is capable of reversing sinusoidal capillarisation and inhibiting hepatic fibrogenesis.

In our study, in cirrhotic rats riociguat opposed the intrahepatic vasoconstriction. One central pathway of NO/cGMP mediated vasodilation is via inhibition of RhoA^43^. RhoA is a small GTPase, which ultimately phosphorylates the motorproteins moesin and myosin and thereby causes vasoconstriction^44^. In BDL rats intrahepatic RhoA is highly upregulated, which contributes to the increased hepatic vascular resistance^45,46^. Here, we noted in RIO-treated BDL animals and also in RIO-treated CCl4 rats a very strong decrease of hepatic phosphorylated moesin and myosin expression, confirming that RIO promotes sinusoidal vasorelaxation, independent of etiology.

RIO ameliorated intrahepatic vascular dysfunction also via other pathways. In line with previous work^24^, we observed that sGC agonism normalized pathological VEGFR2 overexpression and increased expression of eNOS in BDL rats. Yet effects on VEGFR2 were most pronounced in early cholestasis, while RIO increased eNOS expression and activity in advanced BDL. This suggests different effects of sGC agonism during the time course of liver disease. Indeed, there exists a complex and reciprocal interplay between VEGF/VEGFR2 and eNOS/NO in PHT. These expressional changes might be caused by cGMP-mediated increase of dimethylarginine dimethylaminohydrolase^47^, which supports eNOS availability (by clearing asymmetric dimethylarginine (ADMA)) and regulates VEGF expression^48^. Finally both, reduced VEGFR2 activity and increased eNOS activity have been shown to decrease PHT and improve liver fibrosis^11,49^.

Apart from structural and vasoactive effects, we also noted less inflammation in cholestatic livers of BDL rats after RIO treatment, as seen by a decrease of hepatic TNFα expression and monocytes/macrophages infiltration. These observations were more pronounced in rats with advanced BDL cirrhosis, which also presented with decreased liver transaminases after RIO treatment. The anti-inflammatory effects of RIO are in line with other experimental studies, showing that sGC agonism reduces TNFα levels, chemotaxis and thus, leucocyte recruitment in the intestine^50,51^.

Likely both, amelioration of sinusoidal perfusion as well as reduction of intrahepatic inflammation, contribute to the decrease of liver fibrosis and portal pressure in RIO treated animals. Of note, the correlation between fibrogenic/angiogenic biomarkers and changes in portal pressure has also been demonstrated clinically^52^.

While we intended to use various models of PHT in order to overcome model specific limitations, this approach rather revealed etiology-dependent differences in the effects of sGC stimulation on fibrosis and portal pressure. Throughout all experiments, the beneficial effects of RIO were more pronounced in cholestatic cirrhosis than in the CCl4 model. In a small, retrospective clinical investigation, we also noted a higher proportion of HVPG responders to NO donors among patients with cholestatic liver disease as compared to alcoholic/viral etiology. Clearly our observations are limited by a small sample size. However, these findings are supported by data showing that activation of the NO/sGC pathway also stimulates bile secretion^53^, which might be of additional benefit. Moreover, hepatic sGC expression follows a gradient from the portal triad (high expression) towards the central vein (low expression)^38^. While fibrosis in BDL/cholestatic disease mainly originates from the periportal area, in CCl4/alcoholic disease primarily the centrilobular area is affected^54,55^. These distinct pathophysiological characteristics may also explain why RIO is more effective in the cholestatic BDL model as compared to the toxic CCl4 model.

Since RIO is already approved for the treatment of pulmonary hypertension, we also studied the effects of RIO therapy on levels of transaminases in patients. Interestingly, we noted a significant decrease of AST, ALT and alkaline phosphatase during RIO therapy. The clinical significance of this finding is limited by absence of obvious liver disease, yet these effects were only apparent during the RIO treatment period. While pulmonary hypertension and thus, hepatic congestions was improved also in a control group receiving standard medical treatment (without RIO), there were no changes in transaminases over the same time period. This small clinical observation may suggest, that RIO could have hepatoprotective properties also in humans. Of note, RIO has already been tested in patients with liver cirrhosis to assess its safety profile: Besides slower drug excretion and thus increased RIO exposure, no safety concerns were raised in Child B patients, thus encouraging its use also in cirrhotic patients if doses are adjusted to hepatic function^56^.

In conclusion, we demonstrate that sGC stimulation by riociguat ameliorates portal hypertension, reduces liver fibrosis and inhibits hepatic necroinflammation – especially in cholestatic cirrhosis. Our data would suggest that riociguat is most beneficial in patients with compensated (early) biliary cirrhosis, which should be explored in prospective clinical trials.

## Methods

### Ethics

This animal study was approved by the Animal Ethics Committee of the Medical University of Vienna and the Federal Ministry of Science, Research and Economy (BMWFW-66.009/0354-WF/V/3b/2014, BMWFW-66.009/0002-WF/V/3b/2016) and was performed according to the Animal Research: Reporting of *In Vivo* Experiments (ARRIVE) guidelines. Also, the human observations were approved by the Ethics Committee of the Medical University of Vienna (EK-Nr. 2010/796, EK-Nr. 2009/497) and conducted according to the Declaration of Helsinki. Written informed consent was obtained from each patient included in the study.

### Rat models of cirrhosis and portal hypertension

Cholestatic cirrhosis was induced in male Sprague Dawley rats (age 6–8 weeks, 280–330 g) by BDL. BDL animals were maintained for 3 weeks to induce early cirrhosis and for a 5 week duration to induce advanced cirrhosis Fig. 1. Respective controls underwent sham operation (SO).

To induce toxic cirrhosis, rats received iterative intraperitoneal carbon tetrachloride injections (50%v/v CCl4 diluted in olive oil, 4 weeks twice weekly, followed by 4 weeks three times weekly) for a total of 8 weeks. Controls received olive oil (OO). We used the 50%v/v CCl4 solution at a dose of 1 mL/kg to induce early cirrhosis, and 2 mL/kg for development of advanced cirrhosis. According to a sample size calculation (based on the hypothesis that RIO treatment reduces PP by >20%), these four cirrhotic groups comprised 26 animals each, which were randomly assigned.

Non-cirrhotic prehepatic PHT was induced by PPVL using a 20 G blunt-tipped needle as previously described^8^. Healthy controls underwent SO. For this non-cirrhotic group 19 animals were assigned. Hence a total of 123 rats were used for these experiments. All animals received standard pellet chow (V1534, sniff GmbH, Germany), had access to fresh water, were housed in pairs of three in Makrolon cages (T3) with woody litter and followed a 12/12 h light/dark cycle.

### Treatment with riociguat and vehicle

All groups received daily gavage of 1 mg/kg RIO (MedChem Express, Cat.No.: HY-14779, purity: 99.73%, Sollentuna, Sweden) dissolved in VEH (50% dimethyl sulfoxide) or VEH (1 mL/kg) only Fig. 1. The weight-adjusted treatment was administered during the last two weeks in BDL/SO, during the last three weeks in CCl4/OO, and for one week in PPVL animals, respectively.

### Hemodynamic measurements

After completion of treatments, hemodynamic measurements were performed under anaesthesia (ketamine 100 mg/kg; piritramide 2 mg/kg) after a 12 h fasted condition as previously described^9^. Mean arterial pressure (MAP) and heart rate (HR) were recorded after cannulation of the femoral artery (catheter PE-50, Smiths Medical, Kent, UK). Similarly, PP was invasively measured by advancing a catheter through an ileocolic vein. SMABF was measured using a non-constrictive perivascular ultrasonic flowprobe (MA1-PRB, Transonic Systems, Ithaca, NY, USA) placed around the superior mesenteric artery and values were normalized to 100 g bodyweight. All hemodynamic parameters were continuously recorded (ML870 PowerLab 8/30, AD Instruments, Colorado, USA) and analysed using the LabChart7 Pro software. Total portosystemic shunting was calculated by mean relative organ distribution of coloured 15 μm microspheres (DYE-TRAK, Triton Technology, San Diego, USA) after portal venous (red) and splenic (white) injection. After hemodynamic recordings, animals were sacrificed and organs were harvested.

### Fibrosis quantification and biochemical analysis

Detailed descriptions regarding histochemistry, image analysis, Western blotting, PCR, biochemical assays and *in vitro* cell culture experiments are included as Supplementary Methods.

### Human studies

To facilitate translation of the experimental data from bench to bedside two small human studies were conducted. First data of a prospective study including patients with postcapillary PH-HFpEF receiving RIO or standard medical treatment was analysed, regarding their effects on transaminases (AST, ALT), GGT and AP. In a second, retrospective study we compared the effects of nitrates on HVPG between cirrhotic patients with cholestatic versus non-cholestatic liver disease. Detailed descriptions of these two studies are included in the Supplementary Methods.

### Data availability

All data generated or analysed during this study are included in this published article and its Supplementary Information files.

### Statistics

Results are presented as mean ± standard deviation. Distribution of collected values was tested using the Kolmogorov–Smirnov test. Normally-distributed unpaired values were compared using a two-sided student’s t-test. Non-normally-distributed paired values were compared using the Wilcoxon signed-rank test. Fisher’s exact test was applied to assess proportions as it is more accurate with small sample sizes. Primary study outcome parameters were the changes in portal pressure and liver fibrosis. GraphPad PRISM 7 (GraphPad Software Inc, La Jolla, CA, USA) was used for statistical analyses. Two-sided p-values < 0.05 denoted a statistical significance.

## Electronic supplementary material

Supplementary file
