# Supplementary material for: The soluble guanylate cyclase stimulator riociguat reduces fibrogenesis and portal pressure in cirrhotic rats
Source: Sci Rep. 2018 Jun 19;8:9372. doi: 10.1038/s41598-018-27656-y (PMC6008436; doi:10.1038/s41598-018-27656-y)
Supplement: Supplementary file 1 — Supplementary file [file 41598_2018_27656_MOESM1_ESM.docx]

**The soluble guanylate cyclase stimulator riociguat reduces fibrogenesis and portal pressure in cirrhotic rats**

Philipp Schwabl, Ksenia Brusilovskaya, Paul Supper, David Bauer, Philipp Königshofer, Florian Riedl, Hubert Hayden, Claudia D. Fuchs, Judith Stift, Georg Oberhuber, Stefan Aschauer, Diana Bonderman, Thorsten Gnad, Alexander Pfeifer, Frank E. Uschner, Jonel Trebicka, Nataliya Rohr-Udilova, Bruno K. Podesser, Markus Peck-Radosavljevic, Michael Trauner, Thomas Reiberger

**TABLE OF CONTENTS:**

Supplementary methods 2

Fibrosis quantification and biochemical analysis 2

Equipment and settings 6

Human studies 7

References 8

Supplementary Table S1 9

Supplementary Table S2 10

Supplementary Table S3 11

Supplementary Fig. S1 12

Supplementary Fig. S2 13

Supplementary Fig. S3 14

Supplementary Fig. S4 15

Supplementary Fig. S5 17

**SUPPLEMENTARY METHODS**

**FIBROSIS QUANTIFICATION AND BIOCHEMICAL ANALYSIS**

**Immunostaining of liver tissue**. Liver tissue was harvested and immunostained using either the CAB method or antibodies against αSMA, CD68 or CK19. The CAB method was used to assess the degree of fibrosis. 2µm thick slides were prepared from formalin fixed, paraffin embedded whole tissue sections and mounted on superfrost glass slides. CAB staining was prepared as previously described[^1^](#_ENREF_1). For staining of αSMA and CD68, one lobe of the liver was fixed in formalin (4% in PBS) and embedded in paraffin. 2µm thick sections were first deparaffinized in Neo Clear (Merck Millipore) and then rehydrated through decreasing grades of alcohol. Afterwards, endogenous peroxidases were blocked by immersing slides in 2% H_2_O_2_ in pure methanol. No epitope retrieval was required for CD68 detection, whereas for αSMA, epitope retrieval was performed by boiling in 10mM citrate buffer (pH 6). Samples were then subjected to blocking with appropriate serum and with avidin/biotin blocking kit (Vector Laboratories). Subsequently, antibodies directed against CD68 (AbD Serotec, dilution 1:300) or αSMA (Abcam, dilution 1:400) were applied overnight at +4°C or for 1h at room temperature, respectively. Excess antibody was removed by washing with 50mM TRIS-buffered saline. Incubation with secondary antibodies was followed by incubation with Vectastain Elite ABC HRP Kit (Vector Laboratories). Signals were then developed using 3,3′-diaminobenzidine tetrahydrochloride hydrate (DAB, Sigma Aldrich) and nuclei were stained with haematoxylin (Merck Millipore). Immunohistochemistry for CK19 was performed as described previously[^2^](#_ENREF_2). Briefly, liver cryosections were fixed in 4.5% buffered formalin (5 minutes), followed by methanol (5 minutes at −20°C, acetone for 3 minutes at −20°C). CK19 was then identified using the monoclonal rat anti–Troma-III antibody, developed by Rolf Kemler (Max-Planck Institute, Freiburg, Germany). Finally the antibody was detected using the ABC system (Dako, Glostrup, Denmark) with β-amino-9-ethyl-carbazole (Dako) as a substrate. For image analysis, representative fields of view (n=3 per section) were photographed (Olympus BX51 microscope, 4-20x magnification) and positive stained area was measured using image analysis software (ImageJ V1.8, NIH, USA). Examiners were blinded to the respective groups.

**Hydroxyproline.** For measurement of hepatic hydroxyproline content, snap frozen liver tissue pieces (50–100 mg) were homogenized in aqua dest., then precipitated in trichloroacetic acid, followed by three washes in pure ethanol. The protein pellet was hydrolyzed in 6M HCl solution overnight at 95°C. Samples were oxidized with chloramine-T and then incubated with Ehrlich’s perchloric acid solution. Finally, hydroxyproline was quantified by measuring the absorbance at 560nm and the hydroxyproline amount was indicated as µg per g liver tissue.

**Western blotting.** Liver samples were submerged in RIPA buffer with protease- and phosphatase inhibitors and then homogenized in Precellys Lysing Tubes (Bertin, Montigny-le-Bretonneux, France). After quantification of total protein concentration (BCA Protein Assay Kit, Thermo Scientific), 30 μg were separated by SDS polyacrylamide gel electrophoresis (4–20% gradient gels, Lonza, Basel, Switzerland) and subsequently transferred to PVDF membranes (GE Healthcare). After blocking (Western Blocking Reagent, Roche, Penzberg, Germany), incubation with primary antibodies, and secondary HRP-conjugated antibodies (Santa Cruz), the resulting immune complexes were detected with ECL Prime Western Blotting Detection Reagent (GE Healthcare). Protein expression was normalized to glyceraldehyde 3-phosphate dehydrogenase GAPDH (#5174, Cell Signaling). Respective blots were incubated with the following primary antibodies: t-eNOS (N3893, Sigma Aldrich), p-eNOS (#9570, Cell Signaling), t-moesin (sc-6410, Santa Cruz), p-moesin (sc-12895, Santa Cruz), myosin light chain 2 (#3672, Cell Signaling), TNFα (ab66579, Abcam), VEGFR2 (sc-505, Santa Cruz), PDGFβ (sc-7878, Santa Cruz) and αSMA (ab5694, Abcam). Respective bands were cropped, if necessary horizontally flipped to align groups and are shown in the main figures. Full-length blots are presented in Supplementary Figure S5.

**NO_x_ measurements.** Liver samples were homogenized in hypotonic (30mM) PBS using Precellys Lysing Tubes and protein content was determined as performed for Western blotting. After deproteination using Amicon Ultra Centrifugal Filters (Merck Millipore), total NO_x_ content was quantified with the Nitrate/ Nitrite Colorimetric Assay Kit (Cayman Chemical). NO_x_ values were normalized to the sample’s protein content and are shown as μmol / g protein.

**Hepatic gene expression.** RNA from 25 mg frozen liver was isolated using TRIzol™ Reagent (Thermo Fisher Scientific) according to the manufacturer ́s instructions. RNA, which precipitated by adding n-propanol, was washed with 75% ethanol and then resuspended in 50µL of nuclease free water. cDNA was synthesized from 2μg of total RNA using the High Capacity cDNA Reverse Transcription Kit (Thermo Fisher Scientific). RT-PCR was performed using a SYBR Green Master Mix (Thermo Fisher Scientific) and a 384-format ABI 7900HT Sequence Detection System (Applied Biosystems). The following genes were analysed: V-CAM, TNFα, IL1β and MCP1. Specific primer sequences are listed in Supplementary Table S3. RT-PCR was conducted at 95°C for 10 min, followed by 40 cycles of 95°C for 15s and 60°C for 1 min. The relative quantitation of each target gene was performed using the comparative Ct method with GAPDH as housekeeping gene.

**Gene expression of sGC.** Subsets of liver cells were extracted from healthy and BDL (2 weeks) rats, as previously described[^3^](#_ENREF_3)^,^[^4^](#_ENREF_4). Briefly, primary liver cells were isolated in a two-step pronase-collagenase perfusion fractionated by density gradient centrifugation. Afterwards the purified cells were subjected to PCR analysis. sGC subunit expression was analysed using a ABI 7900HT fast real time PCR machine (Thermo Fisher Scientific) with SYBR Green (Roche) and expression was calculated as 2^-dCt, relative to the housekeeping gene TATA box binding protein (TBP). The PCR was conducted at 95°C for 10min, followed by 40 cycles of 15s at 95°C and 1min at 60°C. The used primers are listed in Supplementary Table S3.

**Biochemical assays.** After hemodynamic measurements, plasma samples were drawn via cardiac puncture. AST (IU/mL) and ALT (IU/mL) were measured using the fully automated Cobas c311 analyzer (Roche Diagnostics) with assay kits provided by the manufacturer.

**In vitro LX-2 experiments.** Human LX-2 HSCs were a kind gift from Prof. Jean-Francois Dufour, Bern and were negatively tested for mycoplasm infestation using the MycoAlertTM Mycoplasma Detection Kit (Lonza). Cells were seeded in 6 wells plates (8x10^4^ cells/well) with culture medium (DMEM with FKS and Pen/Strep) and cultured initially for 24h. The next day, cells were treated for 48h with 10 µM of RIO (dissolved in 0.1% DMSO) or vehicle only. Cell medium and treatment solutions were refreshed every 24h. Cells were then harvested and mRNA expression of αSMA was assessed by RT-PCR as described above using a TaqMan gene expression assay (ACTA2: Hs00909449_m1, Thermo Fisher Scientific) according to manufacturer’s recommendations. GAPDH was used as housekeeping gene (Supplementary Table S3).

**EQUIPMENT AND SETTINGS**

For Figures 2A, 2B, 2E, 4E, 5C and 5D photographs of stained liver slides were taken with an Olympus BX51 microscope, Olympus U Plan S-Apo objectives (4x magnification in Figure 2E; 10x in Figures 2A, 2B, 5C and 5D; 20x in Figure 4E) and a 17 megapixel Olympus DP73 digital microscope camera. No filters were used. The acquisition software was cellSens Dimension (V1.7.1, Olympus) installed on a Windows 7 based PC. Pictures were saved in TIFF format with 1600x1200 Pixel, 16.7 million colours and a resolution of 72 dots-per-inch. Positive stained area was measured using ImageJ software (V1.8, NIH, USA) by defining a staining-colour threshold. Per slide three fields of view were photographed (avoiding artefacts, tissue borders, or larger vessels), positive stained area was quantified and the mean of each slide calculated. Groups were compared using a two-sided student’s t-test.

For Figures 3C, 3F, 4C, 5B and Supplementary Figure S2D, after binding antibodies were washed with detection reagent (Amersham^TM^ ECL Plus Western Blotting Detection Solution, GE Healthcare), membranes were developed on films (Amersham Hyperfilm^TM^ ECL, GE Healthcare) for variable durations to achieve optimal exposure. Exposure times are noted on each full-length blot in Supplementary Figure S5. Films were developed with an AGFA CP 1000 film processor (Type 9462/206, AGFA - Healthcare N.V., Belgium) and scanned in grayscale with 600ppi with a HP Scanjet 6300c scanner using the HP Precision Scan Pro software (V2.03, Hewlett-Packard). Pictures were saved in TIFF format with 256 colours and a resolution of 300 dots-per-inch. Intensity of the bands was measured using ImageJ software and corrected to the individual background colour of each film. Respective bands were cropped, if necessary horizontally flipped to align groups and are shown in Figures 3C, 3F, 4C, 5B and Supplementary Figure S2D. Full-length, uncropped blots are presented in Supplementary Figure S5.

**HUMAN STUDIES**

**Patients treated with riociguat.** Patients with PH-HFpEF received add-on RIO treatment (0.5-2mg three doses per day, titrated according to drug label and arterial blood pressure) to standard therapy[^5^](#_ENREF_5) within a prospective, open-label, non-randomized study at the Division of Cardiology at the Medical University of Vienna between 06/2014 and 01/2016 to evaluate predictors of outcome. Here, we performed a post-hoc analysis on the impact of RIO on the time course of AST, ALT, GGT and AP: (i) 2 months before therapy, (ii) at baseline (BL), (iii) 1 month on treatment and (iv) 2 months after treatment ended. We also collected data of an age-matched PH-HFpEF control group before, at baseline and while under standard medical treatment[^5^](#_ENREF_5). Patients with chronic liver disease or baseline AST, ALT, AP or GGT levels above two times the upper limit of normal were excluded.

**Hepatic venous pressure measurement.** Cirrhotic patients admitted at the Division of Gastroenterology and Hepatology at the Medical University of Vienna between 05/2004 and 06/2012 who suffered from PHT (Baseline HVPG>10mmHg) were screened. Those receiving isosorbide mononitrate (ISMN) treatment and undergoing response measurement of the HVPG between one and three months after treatment initiation, were retrospectively assessed. At both time-points, biometrical and serum parameters, as well as hemodynamic data were collected. Patients with alcoholic (ALD) or viral (VIRAL) liver disease were classified as having non-cholestatic PHT. Patients with primary sclerosing cholangitis (PSC), primary biliary cholangitis (PBC) or an autoimmune liver disease (AUTO) were defined as patients with cholestatic PHT. HVPG was measured under fasting conditions, as previously described [^6^](#_ENREF_6) and HVPG response was defined as a relative decrease of more than 10% or below absolute values of 12mmHg.

**REFERENCES**

1 Roque, A. L. Chromotrope aniline blue method of staining Mallory bodies of Laennec's cirrhosis. *Laboratory investigation; a journal of technical methods and pathology* **2**, 15-21 (1953).

2 Fickert, P. *et al.* 24-norUrsodeoxycholic acid is superior to ursodeoxycholic acid in the treatment of sclerosing cholangitis in Mdr2 (Abcb4) knockout mice. *Gastroenterology* **130**, 465-481, doi:10.1053/j.gastro.2005.10.018 (2006).

3 Klein, S. *et al.* Janus-kinase-2 relates directly to portal hypertension and to complications in rodent and human cirrhosis. *Gut* **66**, 145-155, doi:10.1136/gutjnl-2015-309600 (2017).

4 Granzow, M. *et al.* Angiotensin-II type 1 receptor-mediated Janus kinase 2 activation induces liver fibrosis. *Hepatology* **60**, 334-348, doi:10.1002/hep.27117 (2014).

5 Galie, N. *et al.* 2015 ESC/ERS Guidelines for the diagnosis and treatment of pulmonary hypertension: The Joint Task Force for the Diagnosis and Treatment of Pulmonary Hypertension of the European Society of Cardiology (ESC) and the European Respiratory Society (ERS): Endorsed by: Association for European Paediatric and Congenital Cardiology (AEPC), International Society for Heart and Lung Transplantation (ISHLT). *European heart journal* **37**, 67-119, doi:10.1093/eurheartj/ehv317 (2016).

6 Schwabl, P. *et al.* Interferon-free regimens improve portal hypertension and histological necroinflammation in HIV/HCV patients with advanced liver disease. *Alimentary pharmacology & therapeutics* **45**, 139-149, doi:10.1111/apt.13844 (2017).

**Supplementary Table S1. Baseline characteristics of patients with postcapillary pulmonary hypertension and heart failure with preserved ejection fraction.**

| **Parameter** | **Unit** | **Riociguat** | **Standard medical treatment** | **p-Value** |
| --- | --- | --- | --- | --- |
| n |  | 27 | 34 |  |
| Age | years | 78 ± 6 | 76 ± 10 | 0.800 |
| Sex (m/f) | m/f (%m) | 13/14 (48%) | 10/24 (29%) | 0.185 |
| Platelets | G/L | 213 ± 60 | 248 ± 63 | 0.228 |
| Prothrombin time | % | 73 ± 34 | 83 ± 27 | 0.263 |
| INR | ratio | 1.5 ± 0.7 | 1.4 ± 0.7 | 0.733 |
| Creatinine | mg/dL | 1.3 ± 0.4 | 1.2 ± 0.4 | 0.682 |
| Bilirubin | mg/dL | 0.6 ± 0.3 | 0.6 ± 0.3 | 0.713 |
| Albumin | g/L | 41 ± 1.2 | 40 ± 4.6 | 0.623 |
| AST | U/L | 27.6 ± 8.6 | 28.4 ± 15.9 | 0.803 |
| ALT | U/L | 24.0 ± 8.1 | 26.9 ± 17.3 | 0.420 |
| GGT | U/L | 45.1 ± 22.4 | 43.1 ± 24.7 | 0.689 |
| AP | U/L | 87.4 ± 33.8 | 82.5 ± 21.8 | 0.501 |

**Supplementary Table S2. Baseline characteristics patients with non-cholestatic versus cholestatic cirrhosis receiving isosorbide mononitrate therapy.**

|  | **Non-cholestatic PHT**  (ALD/ VIRAL) | | | **Cholestatic PHT**  (PSC/PBC/AUTO) | | | **p-value** |
| --- | --- | --- | --- | --- | --- | --- | --- |
| n | 49 | | | 7 | | |  |
| Age | 52 | ± | 9.7 | 51 | ± | 11.3 | 0.842 |
| Sex (%male) | 40/49 (82%) | | | 2/7 (29%) | | | 0.002 |
| Hemoglobin | 11.67 | ± | 2.14 | 12.40 | ± | 1.73 | 0.392 |
| Platelets | 119 | ± | 96 | 131 | ± | 96 | 0.755 |
| Leucocytes | 5.25 | ± | 2.41 | 5.19 | ± | 3.35 | 0.954 |
| Bilirubin | 1.65 | ± | 1.10 | 2.64 | ± | 0.90 | **0.067** |
| Albumin | 35.99 | ± | 5.68 | 37.36 | ± | 3.52 | 0.539 |
| Prothrombin time | 69.0 | ± | 17.74 | 68.3 | ± | 19.42 | 0.917 |
| INR | 1.25 | ± | 0.15 | 1.26 | ± | 0.23 | 0.885 |
| Creatinine | 0.95 | ± | 0.55 | 0.74 | ± | 0.08 | 0.321 |
| Sodium | 137.2 | ± | 3.40 | 137.0 | ± | 2.94 | 0.857 |
| AST | 57.5 | ± | 36.2 | 71.7 | ± | 45.0 | 0.352 |
| ALT | 43.1 | ± | 40.3 | 53.0 | ± | 29.9 | 0.534 |
| GGT | 172 | ± | 125 | 133 | ± | 42 | 0.417 |
| AP | 123 | ± | 58 | 148 | ± | 72 | 0.326 |
| Varices | 100% | | | 100% | | | 1.000 |
| Child-Pugh score | 6.86 | ± | 1.77 | 6.86 | ± | 1.57 | 0.994 |
| MELD score | 11.12 | ± | 3.49 | 11.77 | ± | 3.10 | 0.643 |
| Heart rate | 74.4 | ± | 12.7 | 79.4 | ± | 9.4 | 0.322 |
| MAP | 99.3 | ± | 19.6 | 92.0 | ± | 9.6 | 0.340 |
| HVPG (baseline) | 19.65 | ± | 4.47 | 21.00 | ± | 2.77 | 0.442 |
| ISMN dose | 51.0 | ± | 17.8 | 60.00 | ± | 20.0 | 0.228 |
| HVPG (under ISMN) | 17.67 | ± | 4.87 | 16.14 | ± | 4.56 | 0.437 |
| ΔHVPG% | -9.87% | | | -22.12% | | | **0.092** |
| % Responder | 43% | | | 86% | | | 0.034 |

**Supplementary Table S3. Primer sequences for PCR**

| **Protein** | **forward** | **reverse** |
| --- | --- | --- |
| VCAM | taa ttg cta tga gga tgg aag act c | gga gat gtc aac aat aaa tgg ttt c |
| TNFα | cat ctt ctc aaa att cga gtg aca a | tgg gag tag aca agg tac aac cc |
| IL1β | caa cca aca agt gat att ctc cat g | gat cca cac tct cca gct gca |
| MCP1 | ggc tgg aga gct aca aga gg | atg tct gga ccc att cct tc |
| sGC-α1 subunit | cca cat caa cac cgg cta at | gaa gtg caa gtt cag tct c |
| sGC-β1 subunit | cgg atg cca cgg tat tgt ct | ctc ctg gct tga cgc aca tt |
| TBP | cac cgt gaa tct tgg ctg taa ac | cgc agt tgt tcg tgg ctc tc |
| GAPDH | agg tcg gtg tga acg gat ttg | tgt aga cca tgt agt tga ggt ca |

**Supplementary Fig. S1. Hepatic NO_x_ levels in BDL rats**


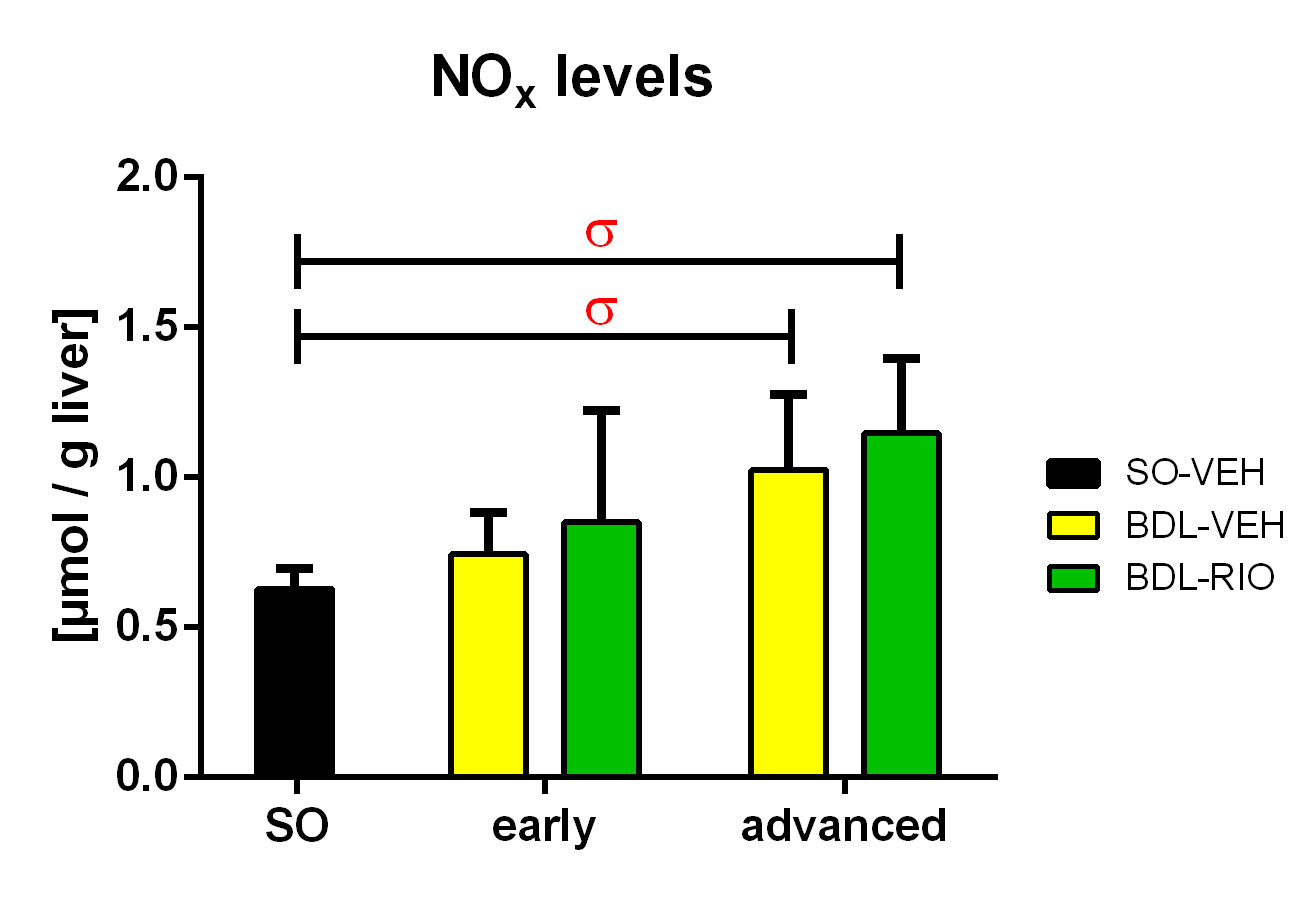


**Supplementary Fig. S1. Hepatic NO_x_ levels in BDL rats.** After liver homogenization, hepatic NO_x_ levels were measured using a nitrate/nitrite colorimetric assay. Total NO_x_ levels increased from healthy to diseased animals and were significantly higher in BDL rats with advanced cholestatic cirrhosis. RIO treatment did not change NO_x_ levels. σ: p<0.05 vs. SO-VEH rats; unpaired t-test; n=5-7 per group - according to Table 1.

**Supplementary Fig. S2. Effects of RIO on protein expression in early CCl4 rats**


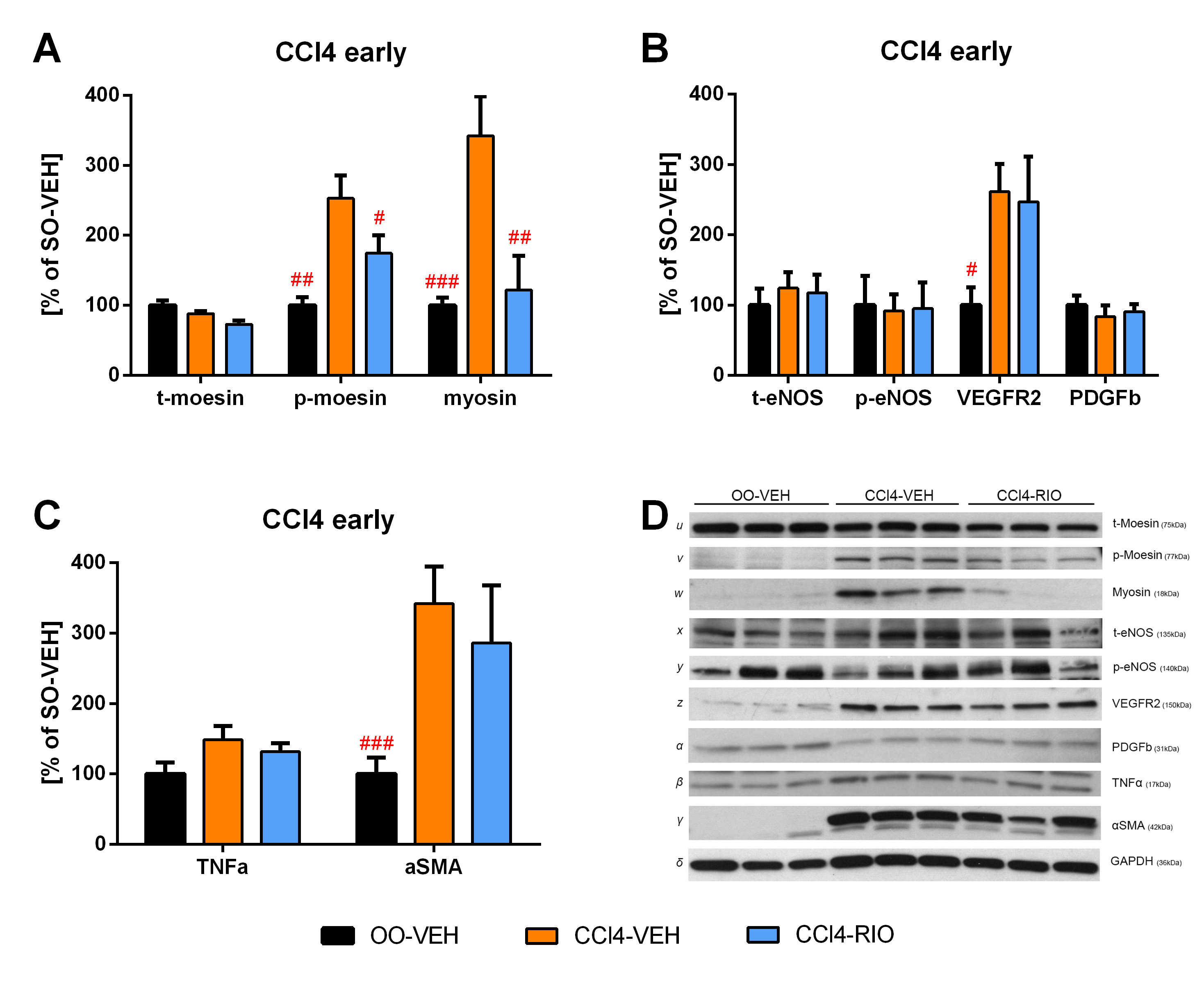


**Supplementary Fig. S2. Effects of RIO on protein expression in early CCl4 rats.** Western blotting was performed to quantify hepatic protein expression. (A) As compared to vehicle treated animals, RIO significantly reduced moesin phosphorylation and total myosin content (thus counter steering vasoconstriction) in rats with early CCl4 fibrosis. (B) CCl4 significantly increased VEGFR2 expression, yet RIO did not affect the intrahepatic protein content of eNOS or growth factors. (C) The livers of CCl4 rats had significantly increased αSMA content. Yet, RIO had no effect on TFNα or αSMA expression. (D) Representative Western blots [u-δ] of CCl4 animals. Full-length blots are presented in Supplementary Figure S5. ^#^p<0.05, ^##^p<0.01, ^###^p<0.001 vs. CCl4-VEH; two-sided unpaired t-test; n=3 animals per group in all panels.

**Supplementary Fig. S3. Effects of riociguat on liver enzymes in non-cirrhotic PH-HFpEF patients.**


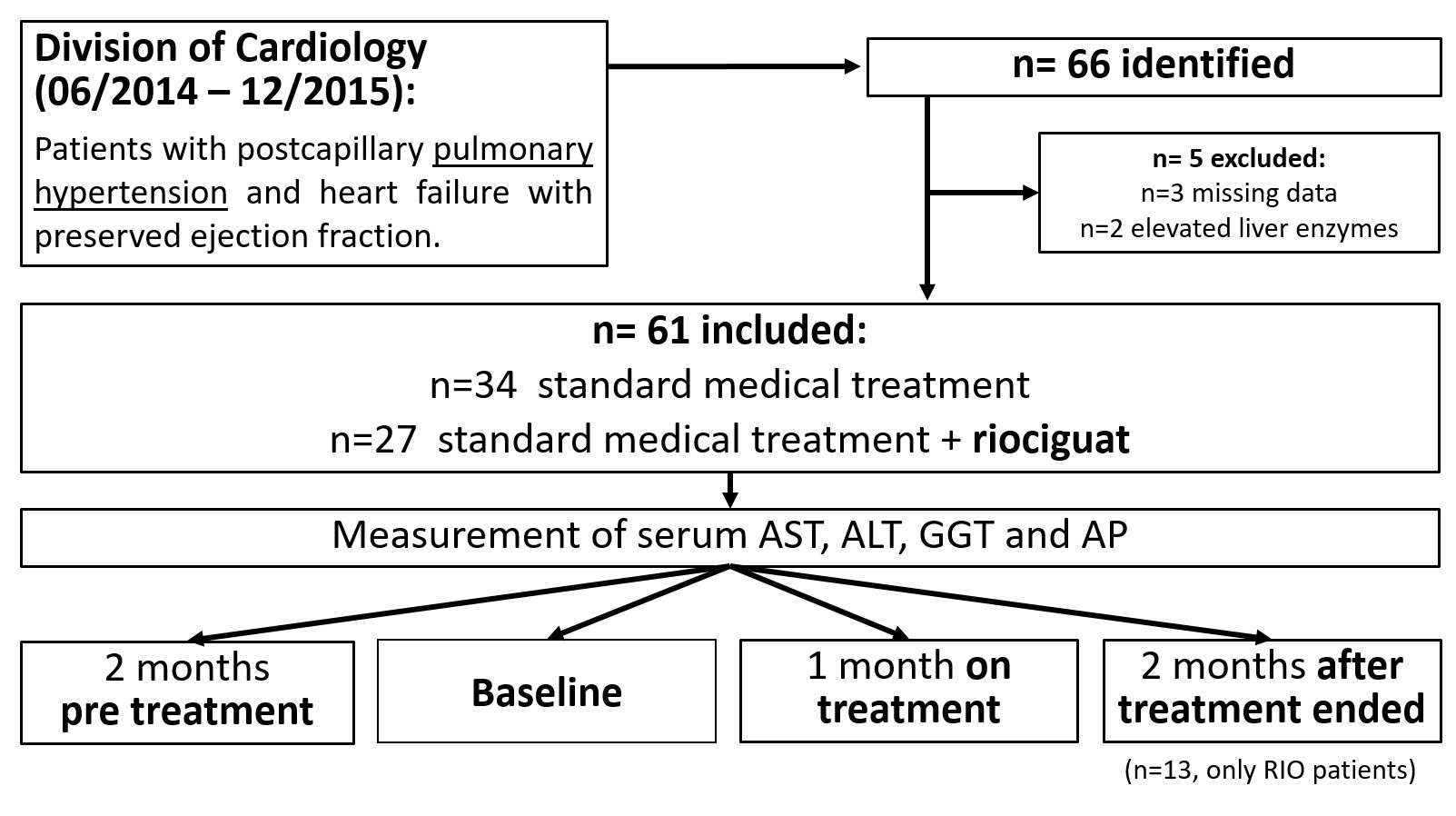


**A**


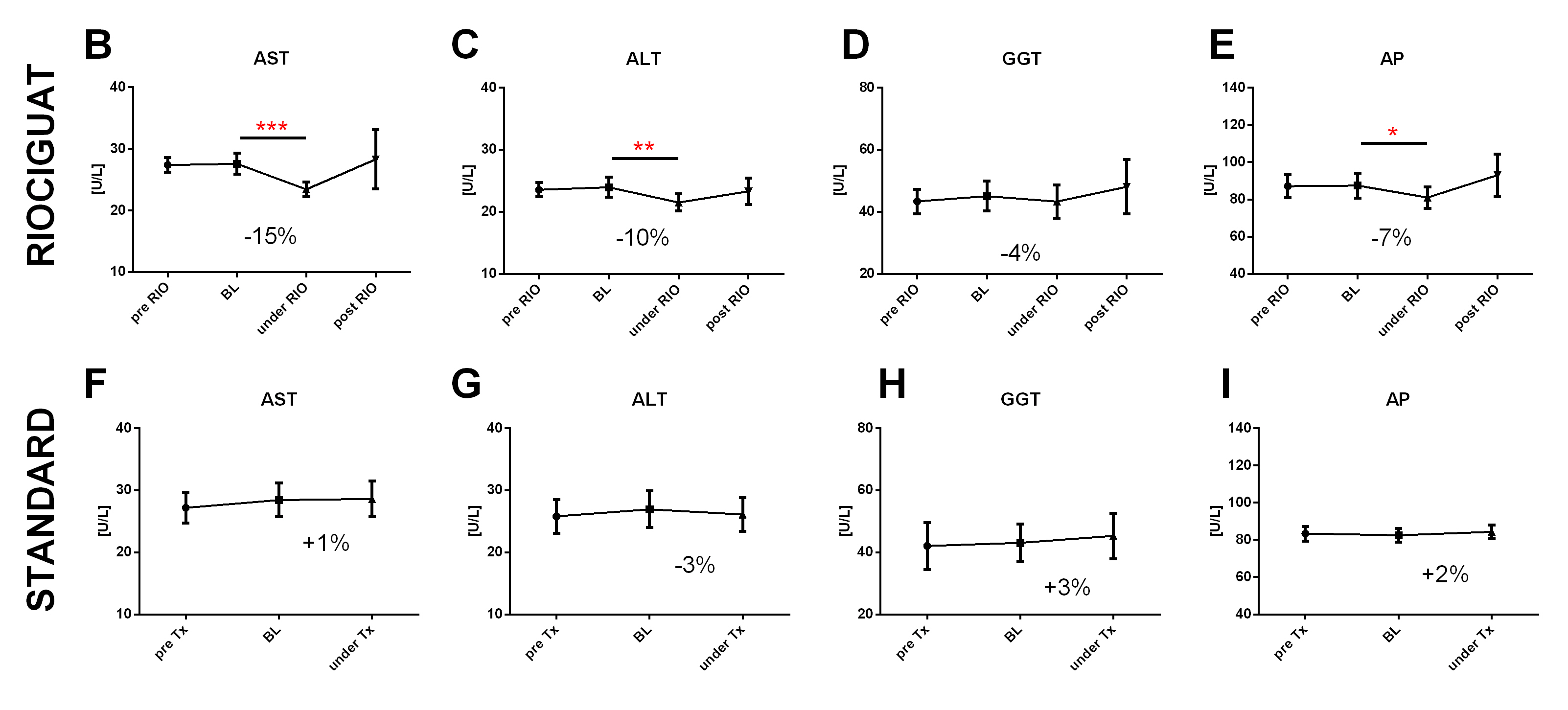


**Supplementary Fig. S3. Effects of riociguat on liver enzymes in non-cirrhotic PH-HFpEF patients.** (A) Scheme of the retrospective study design to analyse the effects of RIO on serum transaminases in non-cirrhotic patients with pulmonary hypertension. The time course of (B) AST, (C) ALT, (D) GGT and (E) AP in non-cirrhotic patients with pulmonary hypertension before, at baseline (BL), under and after therapy with RIO shows a significant decrease of AST, ALT and AP while under RIO treatment. (F-I) In contrast, no changes in transaminases were seen in an age-matched control group receiving standard therapy (Tx). *p<0.05, **p<0.01, ***p<0.001 vs. BL; two-tailed Wilcoxon signed-rank test; n=27 (B-E) and n=34 (F-I).**Supplementary Fig. S4. Patients with portal hypertension receiving isosorbide mononitrate.**


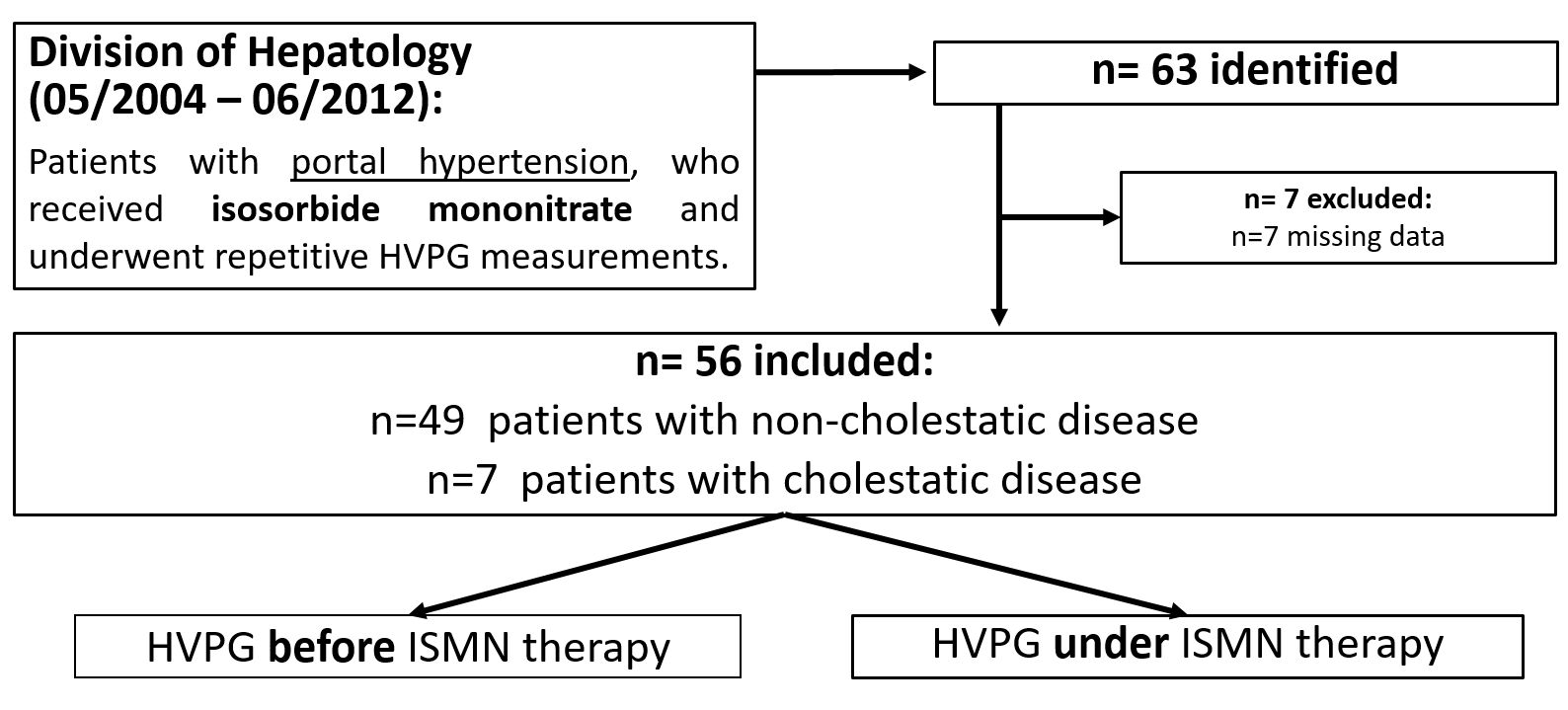
 **A**


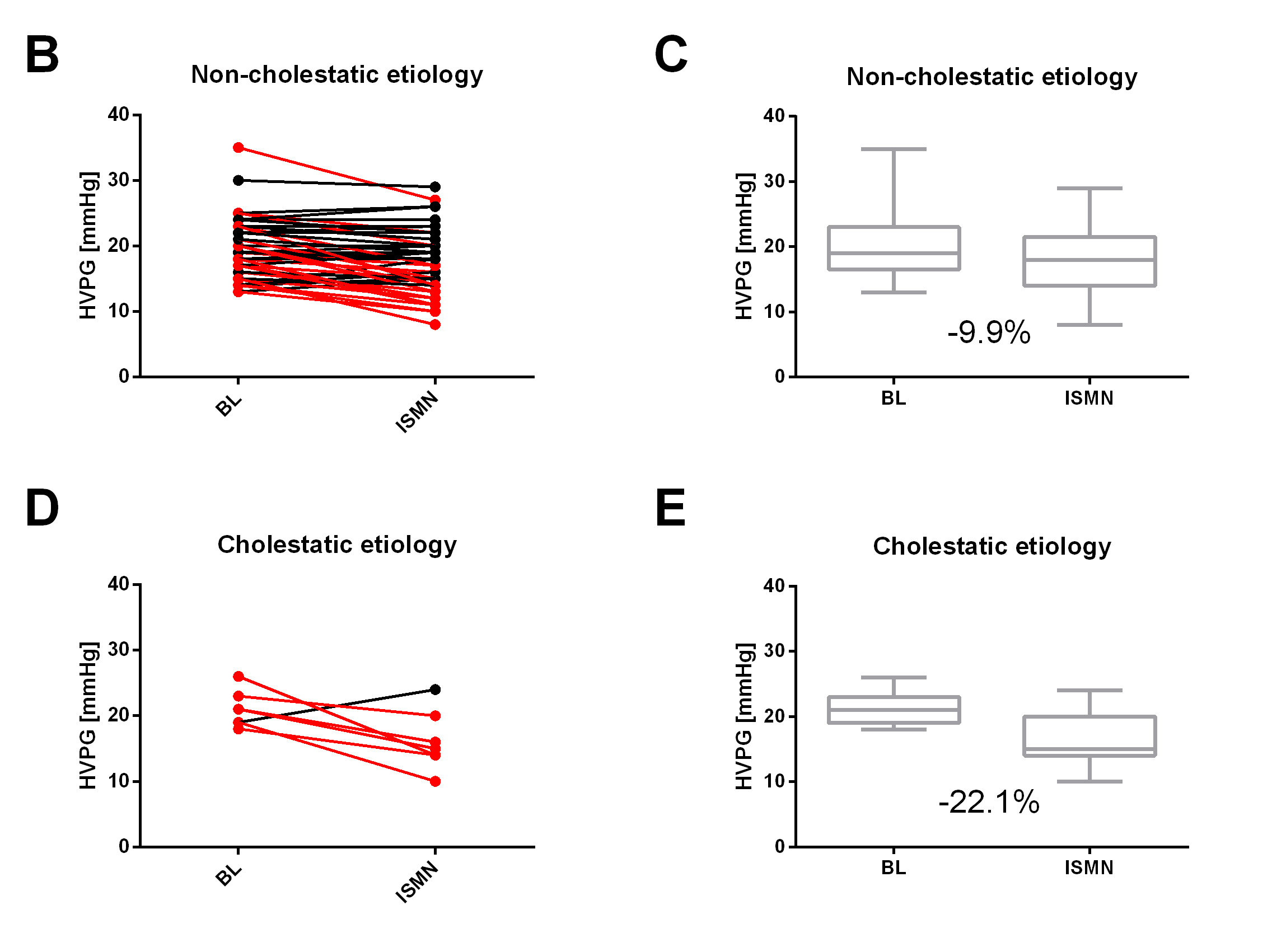


**Supplementary Fig. S4. Patients with portal hypertension receiving isosorbide mononitrate.** (A) Scheme of the retrospective study design to quantify the effects of isosorbide mononitrate (ISMN) treatment on hepatic venous pressure gradient (HVPG) changes in patients with and without cholestatic liver disease. HVPG was measured at baseline (BL) and while under ISMN therapy. (B, C) Patients with non-cholestatic liver disease respond in only 43% to ISMN (red lines), yielding in a mean relative HVPG decrease of -9.9%. (D, E) In patients with cholestatic liver disease the hemodynamic response rate to ISMN was significantly higher with 86%, causing a mean relative HVPG decrease of -22.1%; two-tailed Fisher's exact test; n=7 cholestatic patients and n=49 patients with non-cholestatic liver disease.

**Supplementary Fig. S5. Full-length Western Blots.**


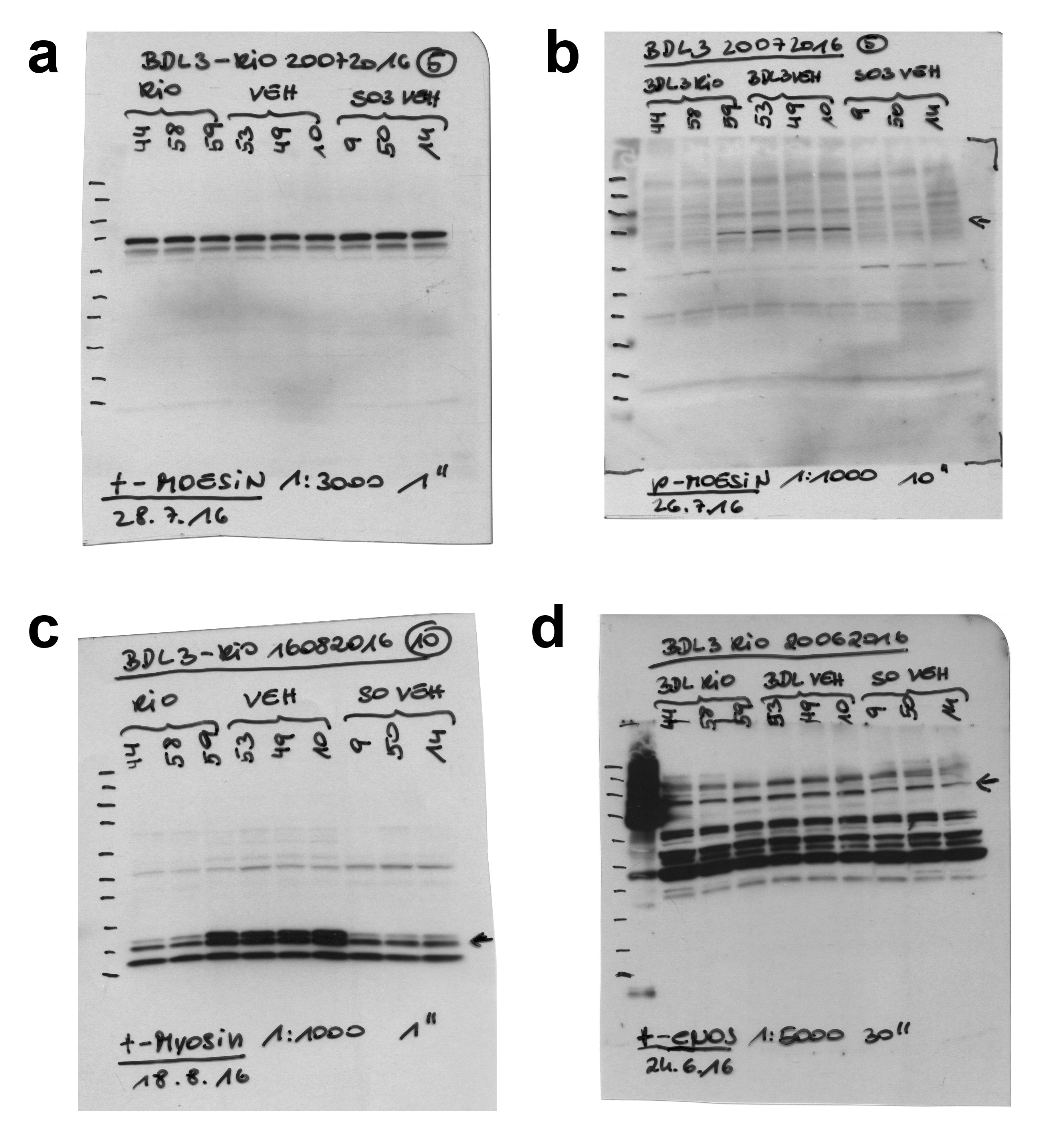


**Supplementary Fig. S5a-d.** Full-length Western Blots of rat livers from early bile-duct ligated animals receiving riociguat (BDL-RIO) or vehicle (BDL-VEH) treatment and respective sham-operated vehicle-fed controls (SO-VEH), showing tissue expression of (A) total moesin, (B) phosphorylated moesin, (C) myosin and (D) total endothelial nitric oxide synthase (eNOS).


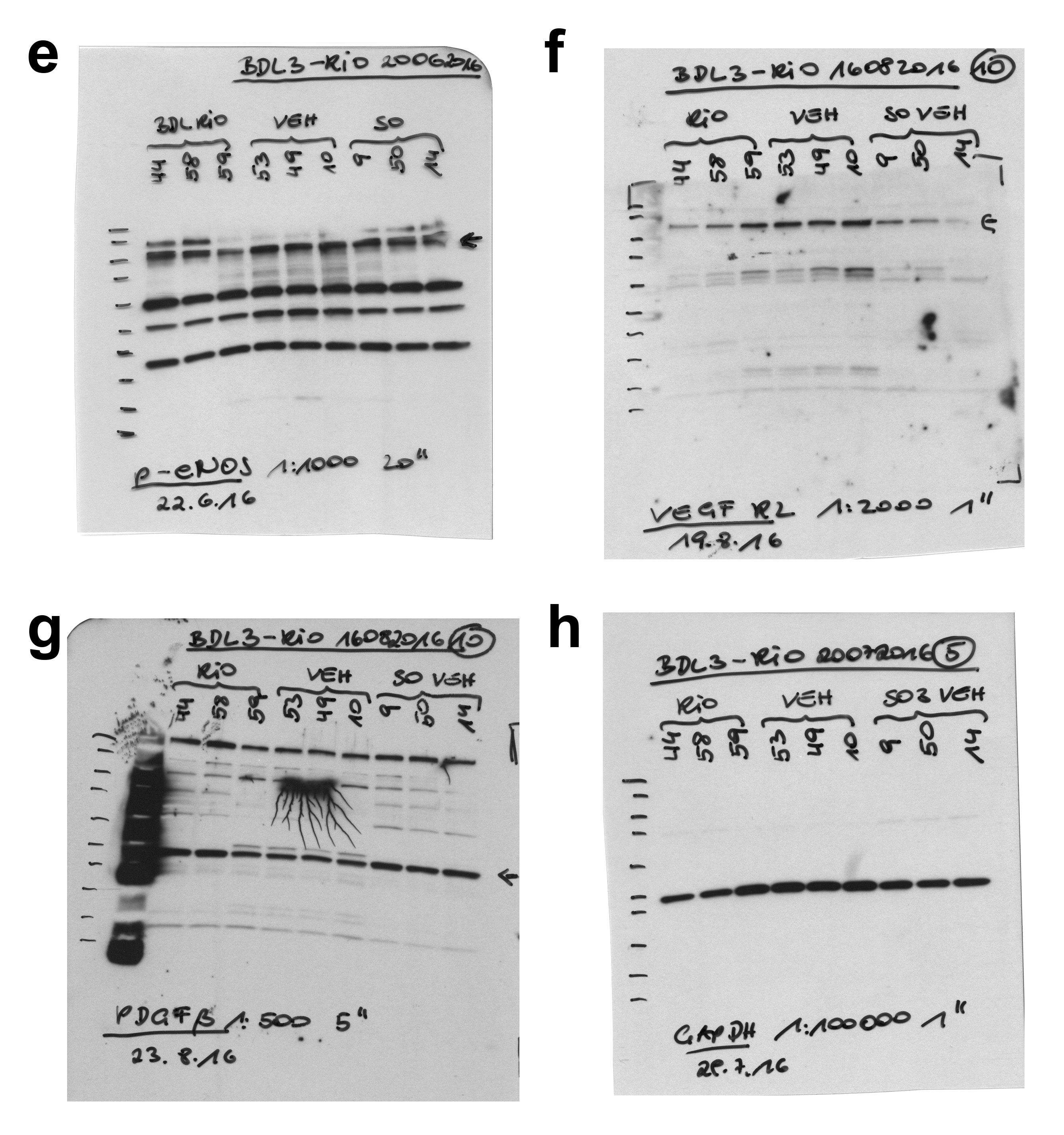


**Supplementary Fig. S5e-h.** Full-length Western Blots of rat livers from early bile-duct ligated animals receiving riociguat (BDL-RIO) or vehicle (BDL-VEH) treatment and respective sham-operated vehicle-fed controls (SO-VEH), showing tissue expression of (E) phosphorylated endothelial nitric oxide synthase (p-eNOS), (F) vascular endothelial growth factor receptor 2 (VEGFR2), (G) platelet-derived growth factor beta (PDGFβ) and (H) glyceraldehyde 3-phosphate dehydrogenase (GAPDH).


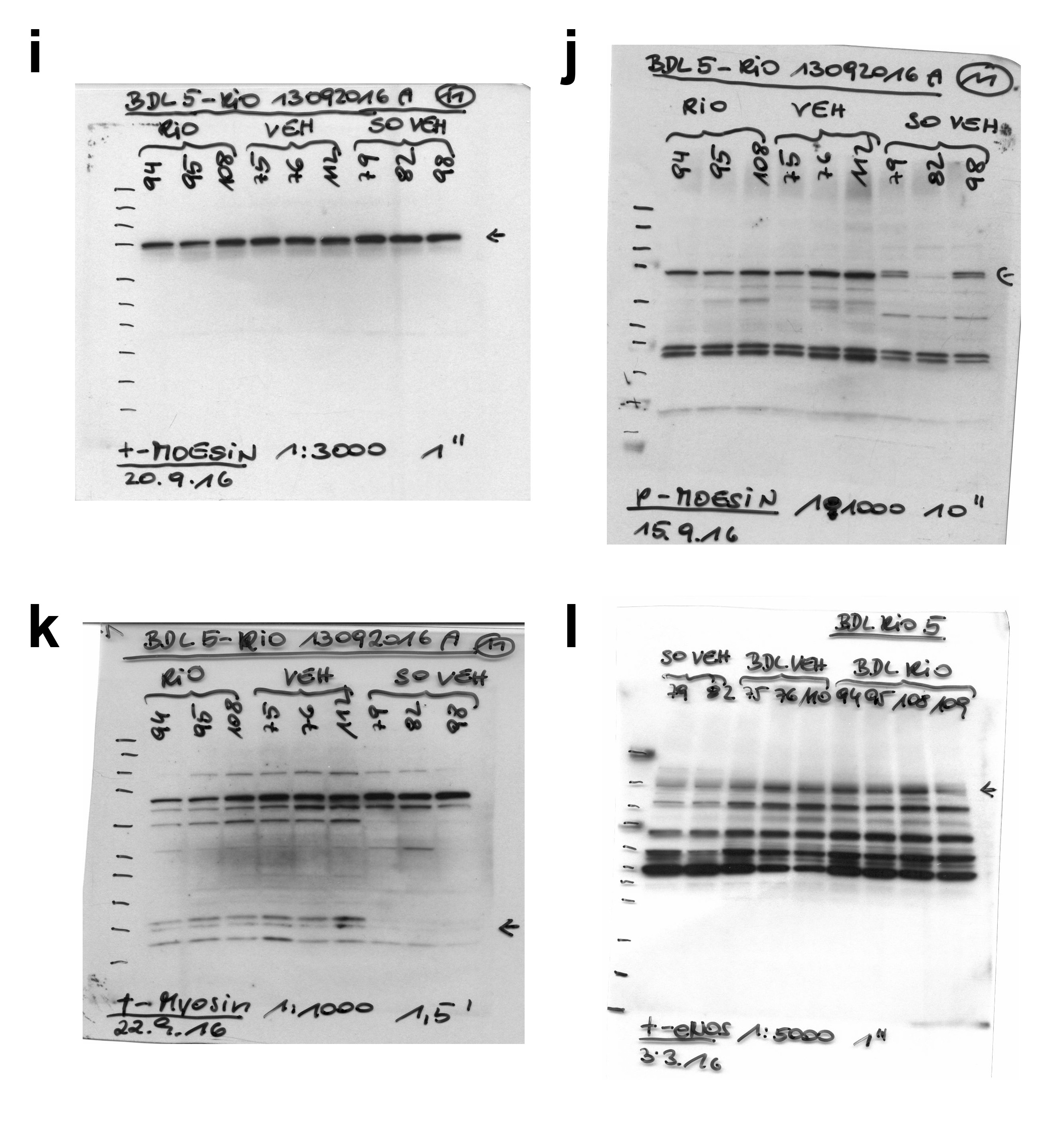


**Supplementary Fig. S5i-l.** Full-length Western Blots of rat livers from advanced bile-duct ligated animals receiving riociguat (BDL-RIO) or vehicle (BDL-VEH) treatment and respective sham-operated vehicle-fed controls (SO-VEH), showing tissue expression of (I) total moesin, (J) phosphorylated moesin, (K) myosin and (L) total endothelial nitric oxide synthase (eNOS).


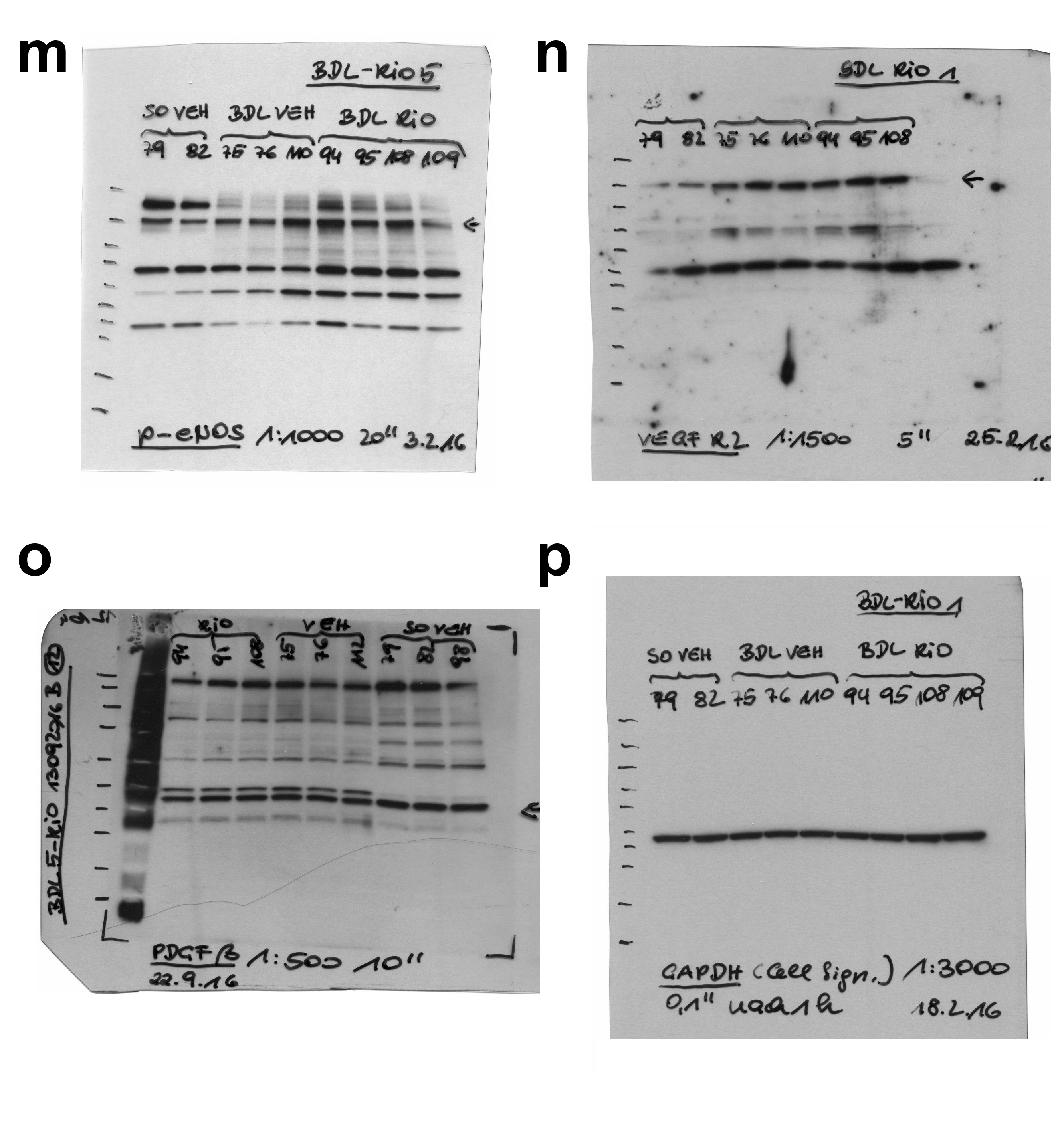


**Supplementary Fig. S5m-p.** Full-length Western Blots of rat livers from advanced bile-duct ligated animals receiving riociguat (BDL-RIO) or vehicle (BDL-VEH) treatment and respective sham-operated vehicle-fed controls (SO-VEH), showing tissue expression of (M) phosphorylated endothelial nitric oxide synthase (p-eNOS), (N) vascular endothelial growth factor receptor 2 (VEGFR2), (O) platelet-derived growth factor beta (PDGFβ) and (P) glyceraldehyde 3-phosphate dehydrogenase (GAPDH).


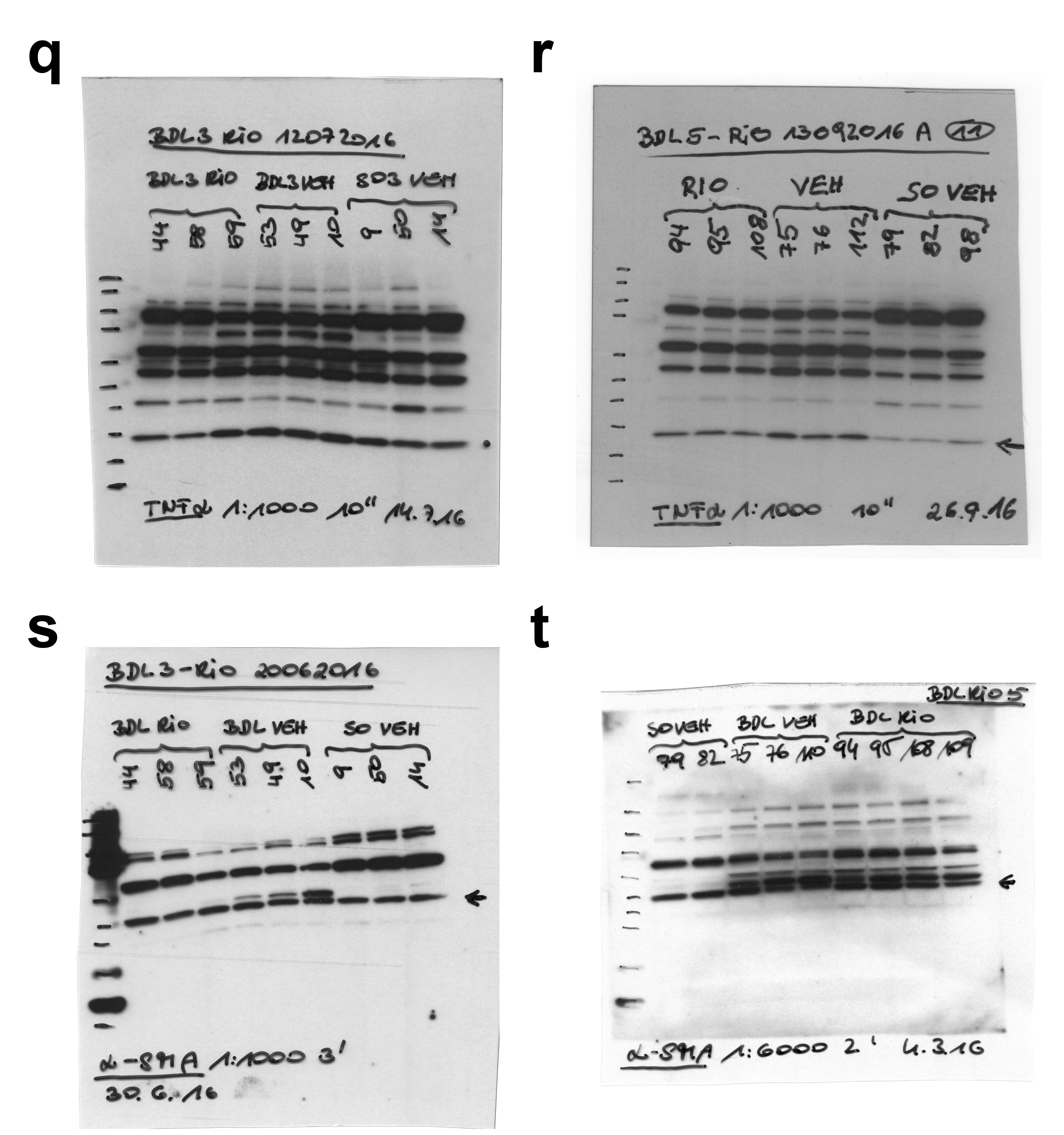


**Supplementary Fig. S5q-t.** Full-length Western Blots of rat livers from bile-duct ligated animals receiving riociguat (BDL-RIO) or vehicle (BDL-VEH) treatment and respective sham-operated vehicle-fed controls (SO-VEH), showing tissue expression of tumor necrosis factor alpha (TNFα) in the (Q) early and (R) advanced bile duct ligation model, and tissue expression of alpha smooth muscle actin (αSMA) in (S) early and (T) advanced BDL.


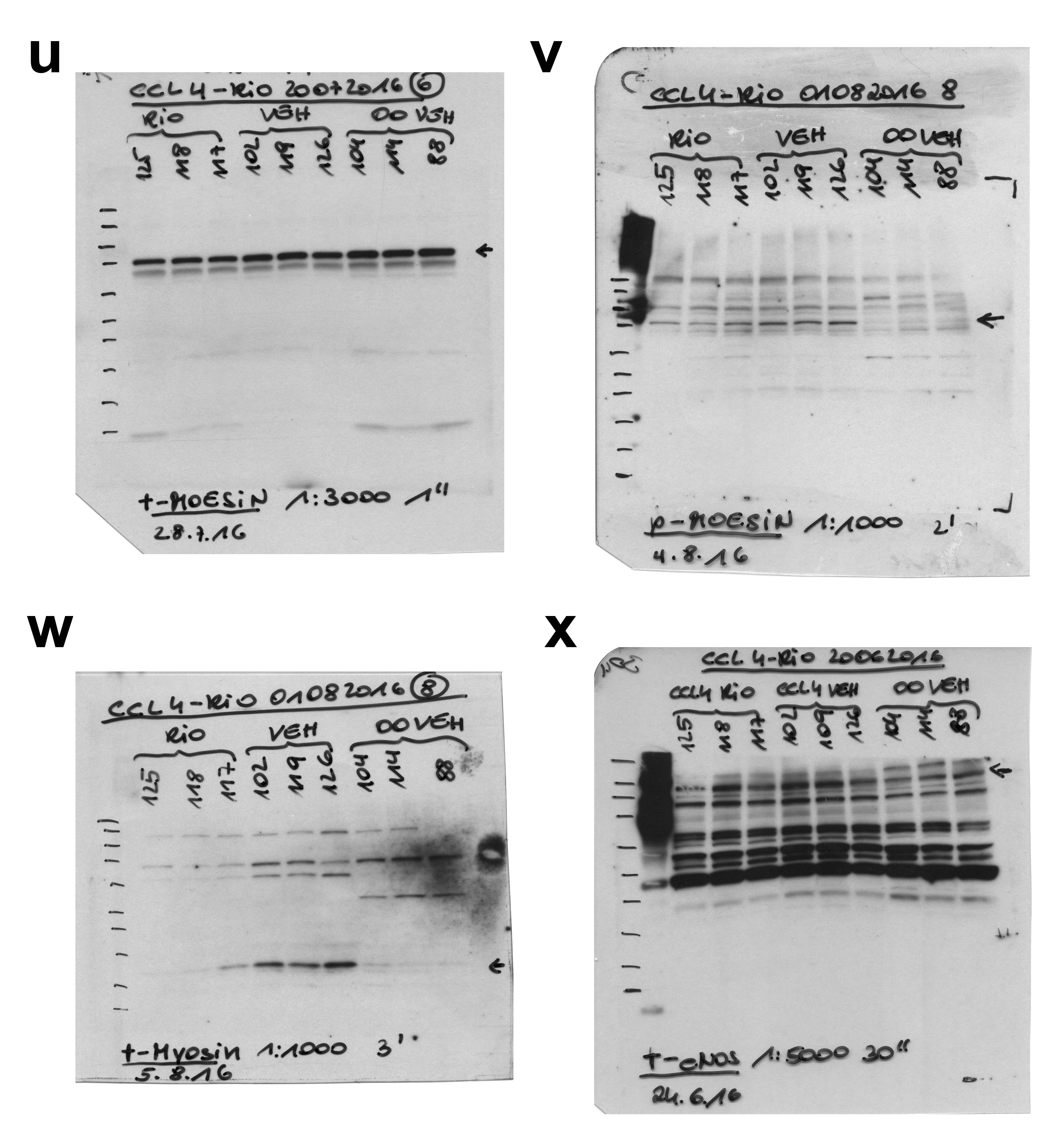


**Supplementary Fig. S5u-x.** Full-length Western Blots of rat livers from early carbon tetrachloride animals receiving riociguat (CCl4-RIO) or vehicle (CCl4-VEH) treatment and respective olive-oil vehicle-fed controls (OO-VEH), showing tissue expression of (U) total moesin, (V) phosphorylated moesin, (W) myosin and (X) total endothelial nitric oxide synthase (eNOS).


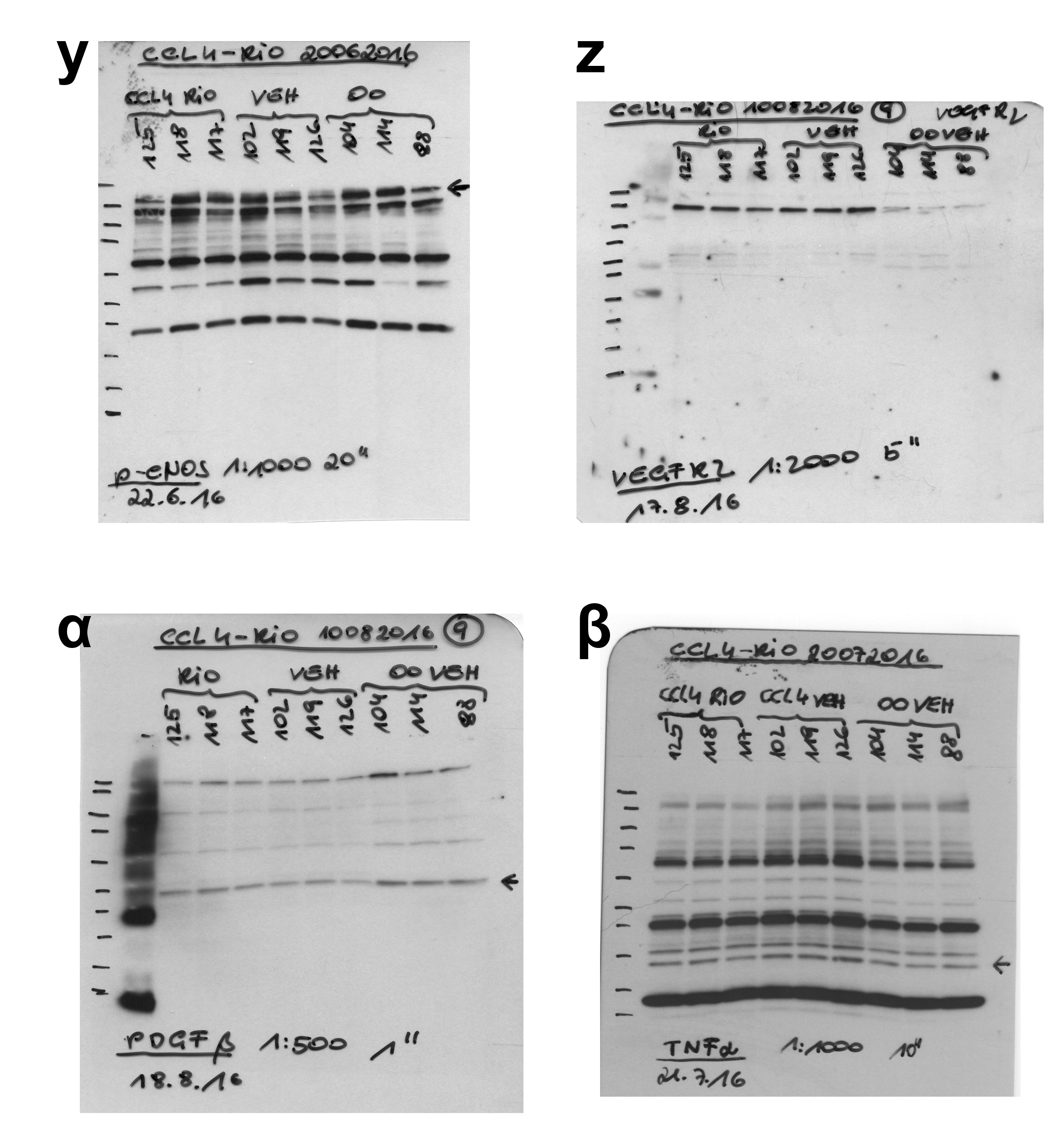


**Supplementary Fig. S5y-β.** Full-length Western Blots of rat livers from early carbon tetrachloride animals receiving riociguat (CCl4-RIO) or vehicle (CCl4-VEH) treatment and respective olive-oil vehicle-fed controls (OO-VEH), showing tissue expression of (Y) phosphorylated endothelial nitric oxide synthase (p-eNOS), (Z) vascular endothelial growth factor receptor 2 (VEGFR2), (α) platelet-derived growth factor beta (PDGFβ) and (β) tumor necrosis factor alpha (TNFα).


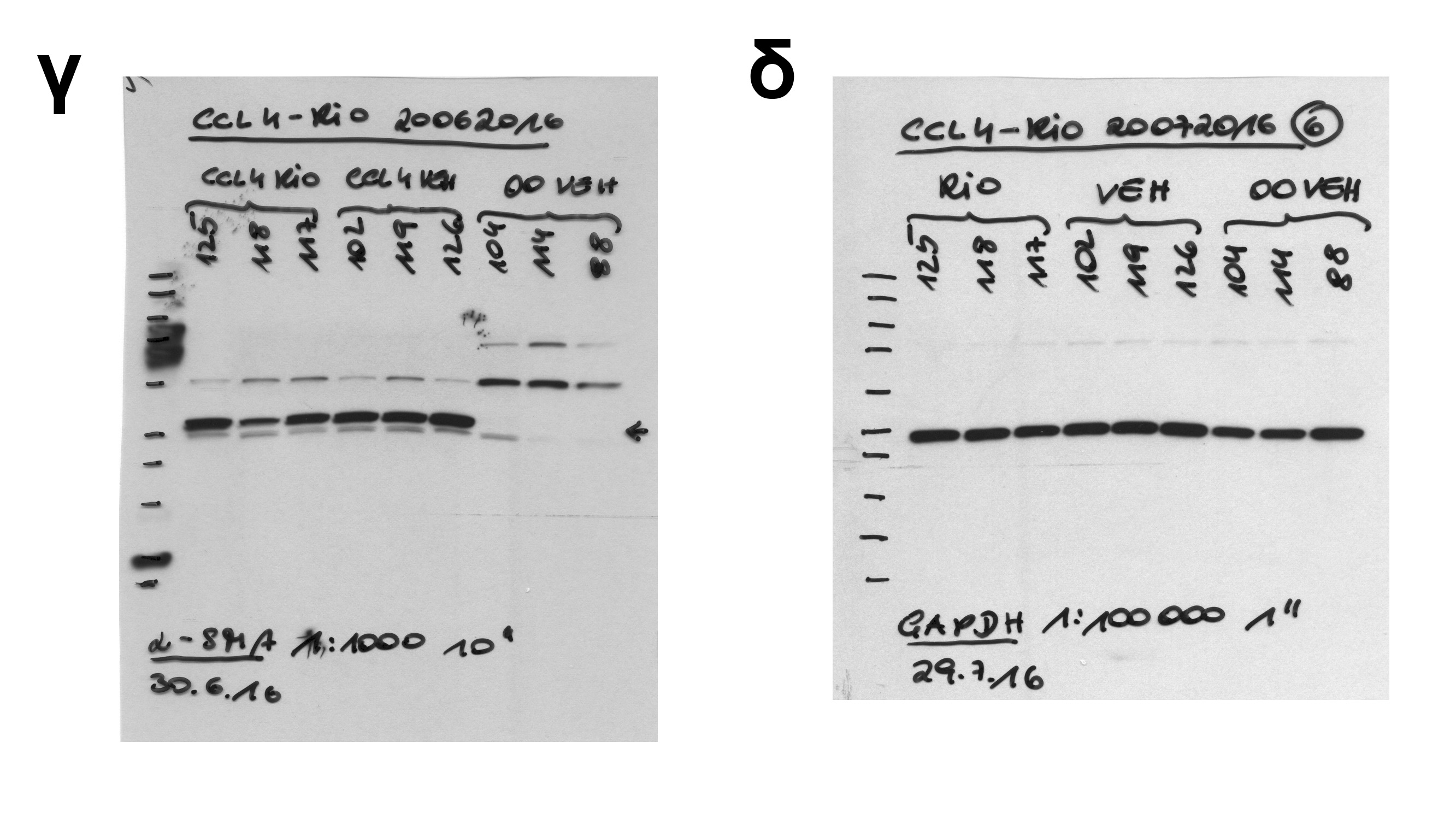


**Supplementary Fig. S5γ-δ.** Full-length Western Blots of rat livers from early carbon tetrachloride animals receiving riociguat (CCl4-RIO) or vehicle (CCl4-VEH) treatment and respective olive-oil vehicle-fed controls (OO-VEH), showing tissue expression of (γ) alpha smooth muscle actin (αSMA) and (δ) glyceraldehyde 3-phosphate dehydrogenase (GAPDH).
